# Maternal mortality ratio and deaths beyond 42 days up to 5-years post pregnancy in women living with HIV on life-long antiretroviral therapy in a resource limited setting

**DOI:** 10.1186/s12884-025-08151-5

**Published:** 2025-11-28

**Authors:** Kerina Duri, Munyaradzi Paul Mapingure, Privilege Tendai Munjoma, Arthur John Mazhandu, Patience Ncube, Tarisai Marere, Mazengera Lovemore Ronald

**Affiliations:** 1 Immunology Unit, Faculty of Medicine and Health Sciences, University of Zimbabwe, UZ-FMHS), P.O. Box A178, Avondale, Harare, Zimbabwe; 2 Community Medicine, UZ-FMHS, Harare, Zimbabwe; 3 Obstetrics and Gynaecology, Unit UZ-FMHS, Harare, Zimbabwe

**Keywords:** Maternal mortality ratio, Woman living with/without HIV, Predictors of maternal mortality, Non-disclosure of HIV sero-status, lifelong antiretroviral therapy

## Abstract

**Background:**

Maternal mortality ratio (MMR) remains poorly described in *w*omen *l*iving *w*ith *H*IV (WLWH), more so during this era of lifelong antiretroviral therapy (ART).

**Methods:**

In a case-control cohort study design with HIV as the exposure variable, we determined MMRs from ≥ 20 weeks’ gestational age up to 42-days postpartum (PP) in WLWH and their peers living without HIV (WLWoH). Furthermore, mortality was assessed 43-days PP up to 5-years following delivery of the index baby. Study midwives administered socio-demographic, HIV-related, household water and sanitation questionnaires in pregnancy. At follow up visits, documented live births, maternal deaths, signs and symptoms of illnesses at death or verbal autopsy, including the place of death as per the Medical Research Council of Zimbabwe’ Serious Adverse Event Reporting requirements. In R-statistical package, using univariate and multivariate automated variable elimination (AVE) models, we assessed the risk factors associated with maternal deaths.

**Results:**

Six-hundred (600) WLWoH, and 608 WLWH mainly on Tenofovir/Lamivudine/Efavirenz (89.3%), ART-naïve (10.4%) and 0.3% ART-defaulters were enrolled. Eighteen maternal deaths were recorded over the 5-years with 56% occurring at home.

Three pregnancy-related fatalities were recorded, all 3 occurring in WLWH; one case each in pregnancy (sepsis), childbirth (obstetric haemorrhage) and within 42-days PP (sepsis); giving a cohort MMR of 3/1202; 249.6 (51.5-727.6)/100000 live births, being 3/602;498.3 (102.9-1449.4)/100000 live births in WLWH. No deaths were recorded in WLWoH within 6-weeks PP while one death was observed well beyond six weeks post-delivery. WLWH were more likely to die compared to WLWoH, odds ratio (OR) 17 (2.29-129.9), *p*=0.006.

Compared with their living peers, mortality in WLWH was associated with a new diagnosis of HIV infection, hence not on ART, *p*=0.027, and non-disclosure of HIV sero-status, *p*=0.014. In AVE regression models, WLWH with plasma HIV-RNA load of more than 1000 copies per mL were more likely to die compared to their alive HIV-RNA suppressed peers, OR 5 (1.2-24.8), *p*=0.03.

**Conclusion:**

WLWH were 17 times more likely to die when compared to WLWoH. Non-disclosure of HIV sero-status was associated with the increased odds of dying, probably due to poor adherence leading to suboptimal ART-exposure. In addition, a new HIV diagnosis, hence being ART-naïve was also a significant contributing factor. There is need to address barriers hindering HIV counselling and testing, treatment, adherence and continuation to suppress HIV-RNA load to reduce MMR in WLWH on lifelong ART.

**Trial registration:**

www.clinicaltrials.gov,trialregistrationnumber:NCT04087239, Registered 12 September 2019.

**Supplementary Information:**

The online version contains supplementary material available at 10.1186/s12884-025-08151-5.

## Introduction

Preventable and treatable pregnancy or childbirth related complications remain the main causes of deaths with a woman dying every two minutes globally, and sub Saharan Africa (SSA) accounted for 70% of all maternal deaths in 2020 [1]. According to the World Health Organisation (WHO), a maternal death is defined as a death of a woman in pregnancy or in 42 days following termination of pregnancy from cause(s) related to or exacerbated by the pregnancy itself or its management but excluding accidental causes [2]. Direct causes include pregnancy induced hypertension, obstetric haemorrhage, anaemia of pregnancy and pregnancy-related sepsis following an abortion or a caesarean section. On the other hand, indirect causes of maternal deaths are those due to pre-existing disease(s) present before conception or health condition(s) that develop during pregnancy not as a result of direct obstetric causes but may get worse due to the physiological effects of pregnancy [2]. Pregnancy as a condition increases the risk of acquiring new infections such as malaria, HIV/AIDS, and the associated opportunistic (co)infections such as tuberculosis (TB) infection, *Pneumocystis jirovecii* pneumonias, and compromises the control of existing infections [3].

The life-time risk of a maternal death of a woman is 1: 5300 live births in high income countries, against 1: 49 in resource limited settings, a stark contrast of the existing inequalities in access to quality health care services [1]. Maternal mortality ratio (MMR), defined as pregnancy or childbirth-related deaths measured per 100,000 live births is an important measure of human and social development, including access to quality health care [4]. MMR is a barometer gauging the functioning status of a country’s health delivery system, and at the same time being an indicator of the overall general health of a population [4].

In Zimbabwe, from 1988 to 1997 the MMRs increased from 50/100,000 to 224/100,000 with puerperal sepsis having been the main cause of maternal deaths [5]. The advent of HIV/AIDS brought about an increase in HIV-related deaths mostly due to meningitis, pneumonias including TB [5]. Mortality surveys conducted in 11 districts in Zimbabwe that investigated the potential causes of maternal deaths in 2007–2008, and between 2018 and 2019 showed that both the direct/obstetric-related, and the indirect maternal deaths decreased by 84% and 61%, respectively, and during the same period MMRs declined from 657/100,000 to 217/100,000 live births [6]. The observed declining MMR trend was partly due to the universal access to ART from the year 2013, complemented by the brief economic stability mainly due to the availability of considerable external health funding [7]. In addition, the decreasing MMR figure was partly as the result of the successful implementation of the WHO strategies that ensured that each pregnant woman received antenatal care, was assisted by a skilled birth attendant during delivery, and that all mother-infant dyads received postnatal care within two days of birth [8]. However, the recent national census statistics for 2022 showed the MMR to be on the increasing trajectory at 363/100,000 live births against a national target of 174/100,000 live births [9]. This is happening in a hyperinflationary environment where extreme poverty of < US$1.8 per day rose from 23% in 2011 to 38% in 2019, amidst a shrinking gross domestic product [10]. Climate change catastrophes, social or civil unrests, the recent covid-19 pandemic, excessive brain drainage of health professionals have exacerbated this dire situation in a country where the HIV prevalence is 11.6% in adults, and stands at 1.8% among children less than 14 years old, with women having the highest prevalence of 14.7% [11].

Population group tailored interventions to specifically meet the needs and addressing challenges faced by women of reproductive age are essential if the 2016–2030 global Sustainable Development Goal (SGD) Target 3.1 [12], aiming to decrease the MMR to less than 70 maternal deaths per 100,000 live births is to be met. MMR remains poorly described in young *w*omen *l*iving *w*ith *H*IV (WLWH) more so in this era of universal access to ART. Previous local studies reported on pooled MMRs that combined statistics of both the WLWH and *w*omen *l*iving *w*ith*o*ut *H*IV (WLWoH). This may be misleading or not be as informative to policy makers as modifiable factors to reduce MMR may differ by maternal HIV status. Furthermore, contemporary evidence has been based on data collected mainly from civil registration, vital statistics and health system records [7], and such results may be underestimations as not all deaths and live births are captured in resource limited settings. In this study there was intense follow up especially up to 6 weeks PP to mitigate MMR underestimations.

### Objectives

We aimed to determine the;

1. MMRs from ≥ 20 weeks’ gestational age through to childbirth until 42 days PP, and describe the potential causes of direct and indirect maternal deaths at 43 days up to 5-years post pregnancy in WLWH and WLWoH enrolled in the University of Zimbabwe Birth Cohort Study (UZBCS).
2. Predictors of maternal deaths in the WLWH subgroup by comparing in retrospect the antenatal factors of the deceased women against those of their living peers.

### Secondary objectives

Compare mortality patterns prior to, during and post the covid-19 era in the UZBCS.

## Method

### Study setting

#### Design of UZBCS

The UZBCS is a prospective non-interventional cohort of WLWH and WLWoH enrolled in pregnancy ≥ 20 weeks’ gestational age from the south-western high-density suburbs of the city of Harare in Zimbabwe. Women were invited to participate in this study when they were seeking antenatal care services at Municipal primary health polyclinics between 2016 and 2019 as previously described [13]. UZBCS aimed to compare the health of infants born to WLWH and WLWoH from birth, and followed up as mother-baby pairs from week(s) 1, 6, 10, 14, 24, 48, 72 and 96 of age, and thereafter seen once a year until five years of age.

Zimbabwean national guidelines stipulate that all WLWH should be on lifelong ART. However, in real practice pregnant women rarely receive their HIV diagnosis in a timely manner. For the prevention of vertical transmission of HIV, pregnant WLWH received (non)–nucleoside reverse transcriptase inhibitors; TENOLAM-E (*Teno*fovir, *La*mivudine and *E*favirenz) since 2013, a standard of care which was gradually replaced with TELOLAM-D (Dolutegravir) since August 2019.

Within the WLWH in the UZBCS, plasma HIV-RNA non-suppression is not uncommon mainly due to first-time HIV diagnosis late in pregnancy, hence not yet on ART or having suboptimal exposures of HIV medications [14]. Antenatal plasma cytomegalovirus DNA > 50 copies/mL has been demonstrated to be an independent risk factor for vertical transmission of HIV [15].

#### Criteria for inclusion and exclusion of research participants into the study

Being pregnant and ≥ 20 weeks of gestational age, 15-years of age at enrolment, and planning to deliver at any of the selected four study sites were the inclusion criteria. The presence of mental health disorders that hindered the research participant to provide informed consent and/or comply with study procedures constituted the exclusion criterion [13].

#### Study procedures

At each study visit questions on socio-economic status, diet including household environment were asked by the study midwife, who also performed full physical examinations. Anthropometry indicators of maternal nutritional status included mid upper arm circumference (MUAC) and body mass index (BMI) as previously described [13].

Additional questions specifically for WLWH, included the date of ART commencement, presence of stock outs, ART adherence, including HIV infection disclosure status; Enrolment questionnaire (Supplementary material). Sample collection, HIV testing, full blood counts, CD4 + T-lymphocytes counts enumeration including HIV-RNA load testing were done as previously described [13]. WLWoH were continuously counselled at each study visit, re-tested for HIV infection every 6 months.

#### MMR data collection

Study midwives trained on the study protocol with valid good clinical practising certificates, and supervised by the study investigators documented all live births and maternal deaths. Dates of maternal deaths were recorded, including the place where the death occurred as per the Medical Research of Zimbabwe’ Serious Adverse Event Reporting requirements; form (ZWE-39). https://clinregs.niaid.nih.gov/updates/full/158-zimbabwe-profile-updated. Maternal signs and symptoms of illness at the time of death or verbal autopsy including the duration of illness or presence of any event(s) preceding the death from the surviving spouse or close relative were documented in cases where the clinical records were not available. The study Obstetrician-Gynaecologist did the classification of direct or indirect maternal deaths.

#### Data management

A secure web-based Research Electronic Data Capture (REDCap v 8.0, © 2020) was used to enter and manage data. Quality assurance strategies to ensure accuracy of data entry included blinded double entries and verification in cases of discrepancies.

#### Data analysis

Maternal deaths were enumerated. Calculations were done for descriptive summary statistics to determine the predictor and outcome variables of interest. Frequencies and percentages were used for categorical variables whilst medians including interquartile ranges (IQR) were used for continuous variables. Testing for normalcy was done using the Shapiro-Wilk test.

In univariate analyses, women who died were compared with their living counterparts with respect to the enrolment socio-demographic characteristics, presence of other comorbidities including spouse/intimate partner factors. Deaths not traditionally considered to be unnatural, such as those due to accidents were excluded from further analyses. In other univariate analyses, each WLWH who died was compared with 4 randomly selected living counterparts with respect to, the presence of other comorbidities, spouse/intimate partner factors, including HIV-related factors such as presence of stigma, timing of ART initiation and adherence, plasma HIV-RNA load including CD4 + T-lymphocytes counts, using the, Mann-Whitney U test, Kruskal Wallis test or Fisher’s exact test where appropriate.

To estimate the odds ratios (ORs) and the 95% confidence intervals (CI) of dying, multivariable logistic regression analyses were performed. For predictors of dying in WLWH, if the death cases would be too few, a subsample of cases and controls was used as a strategy to avoid the pitfalls of overpowered studies. In this study, we employed Automated Variable Elimination (AVE) using the R statistical package to streamline our predictive models by systematically reducing the number of input variables. This automated process involved assessing the significance of each variable, and iteratively removing those with the least contribution to model performance. By focusing on the most relevant factors aiming to enhance model interpretability and efficiency while minimising the risk of overfitting. The resulting simplified models maintained high predictive power, making them robust and suitable for practical applications in our research questions [16]. A *p*-value of less than 0.05 was considered significant. Kaplan-Meier survival estimates and functions were tested using the Log rank test. Statistical analyses were performed in STATA Version 18, Texas USA and R Studio, version 4.1.1.

#### Ethical approval and consent to participate

The study complied with the international council for harmonisation of Good Clinical and Laboratory Practice guidelines including Zimbabwean research regulatory requirements. Institutional ethical approval was obtained from the Joint Research Ethics Committee of the University of Zimbabwe, Faculty of Medicine and Health Sciences/The Parirenyatwa Group of Hospitals (JREC); reference number; JREC/81/15 and ethics approval at the national level was approved by the Medical Research Council of Zimbabwe (MRCZ) reference number MRCZ/A/1968. All research participants all provided written informed consent. Consent form; https://classic.clinicaltrials.gov/ProvidedDocs/39/NCT04087239/ICF_000.pdf.

## Results

### Overall mortality in the UZBCS

**Fig. 1:**
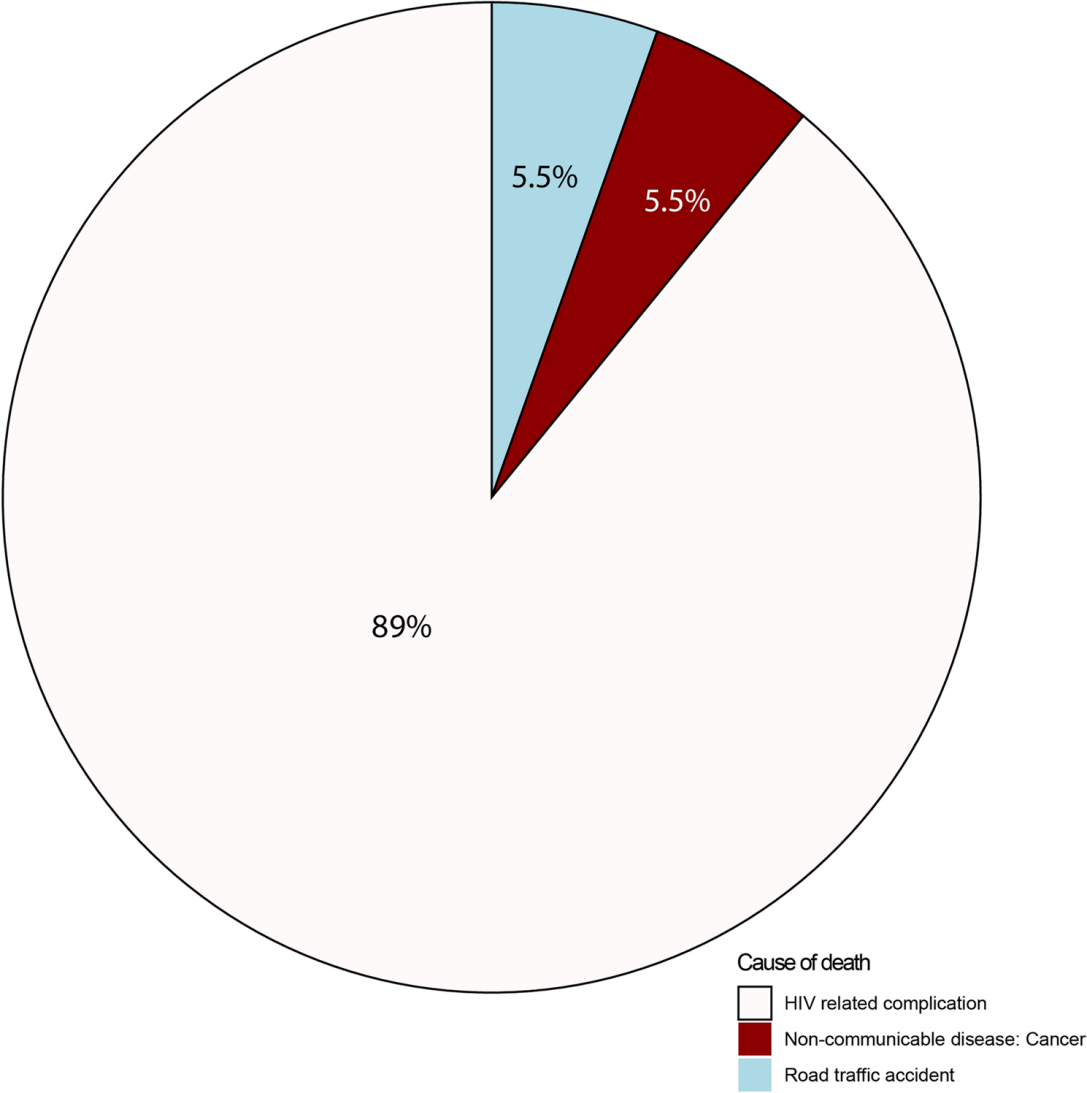
Causes of death in women of reproductive age participating in the University of Zimbabwe Birth Cohort Study (excluding the road traffic accident case)

One thousand two hundred and eight (1208) pregnant women ≥ 20 weeks’ gestational age were recruited between January 2016 - August 2019. Of these 600 were WLWoH, and 608 WLWH mainly on Tenofovir/Lamivudine/Efavirenz combination therapy (89.3%), ART naïve (10.4%) whilst 0.3% had discontinued ART. A total of 18 maternal deaths were observed over the 5-years, thus an overall mortality frequency of 1.4%; being 2.6% and 0.17% in WLWH and WLWoH, respectively. Majority of the deaths (55.6%) occurred at home, and 94.4% were WLWH, *p* = 0.001, Table 1. The main (89%) causes of deaths were HIV-related complications, particularly TB coinfections; Fig. 1.

**Table 1:** Timing and characterisation of mortality in the cohort in women living without HIV (WLWoH) (*n* = 1) versus women living with HIV (WLWH) (*n* = 17)

Variable	Total number of deaths *N* = 18	Death in WLWH (*n* = 17)	Death in WLWoH (*n* = 1)	*p*-Value
Timing of deaths				
In pregnancy up to 42 days	3 (16.7)	3 (17.6)	0 (0.0)	
From 42 days to 5 years	15 (83.3)	14 (82.4)	1 (100.0)	0.645
Cause of death				
HIV related complication				
NCDs	16 (88.8)	16 (94.1)	0 (0)	
RTA	1 (5.6)	0 (0.)	1 (100.0)	**0.001**
	1 (5.6)	1 (5.9)	0 (0)	
Place of death				
Home	10 (55.6)	8 (47.1)	0 (0)	
Health facility	8 (44.4)	9 (52.9)	1 (100.0)	0.357

Data are expressed as n (%) or median (*IQR*); min-max unless stated otherwise. Statistical analysis: comparison of the 2 groups using the Kruskal Wallis test, Mann-Whitney U test or Fisher’s exact test where appropriate. *P*-values in bold font are statistically significant

*NCDs*: Non Communicable Diseases

*RTA*: Road Traffic Accident

**Fig. 2:**
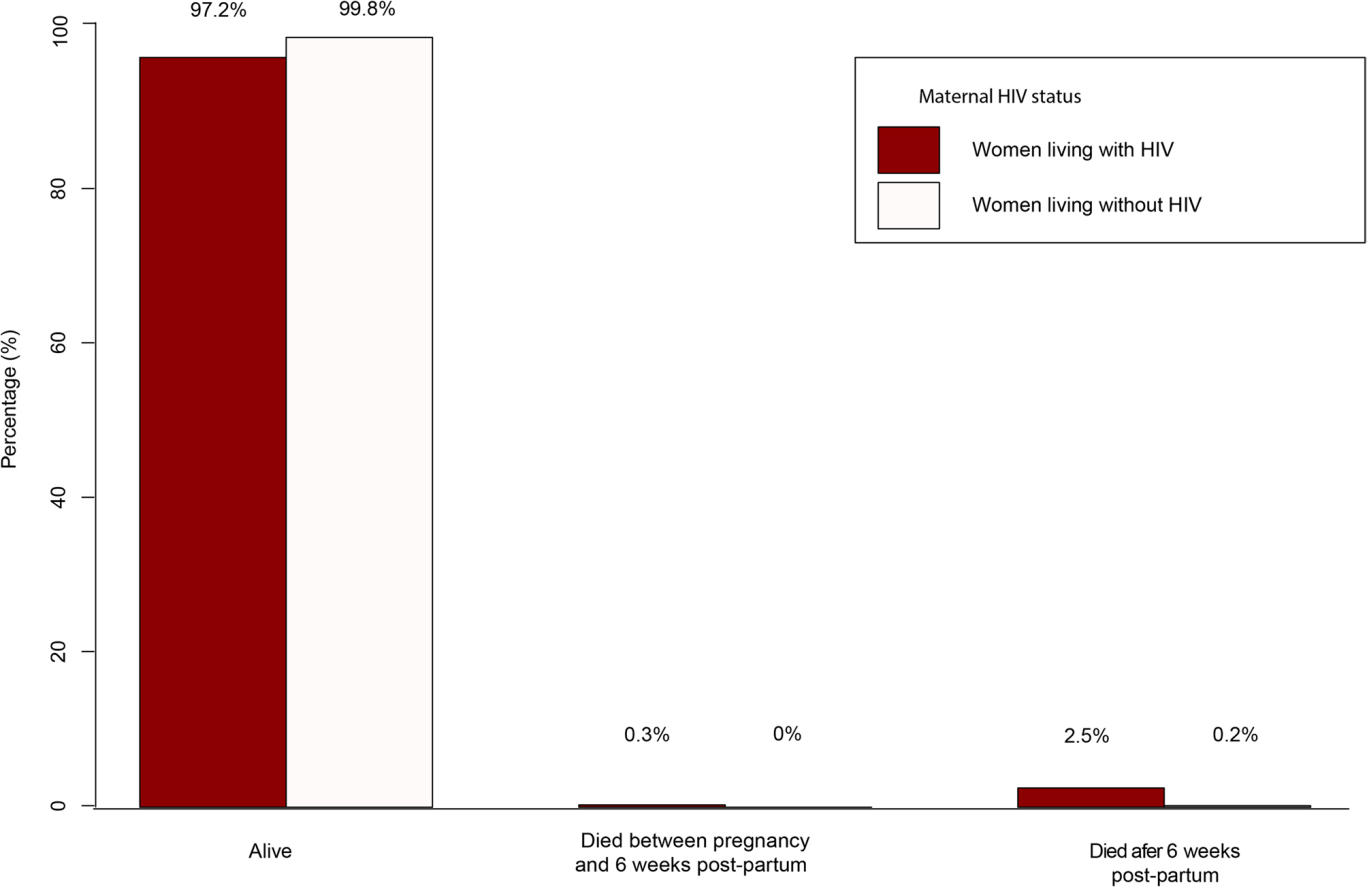
Bar graph showing the proportions of maternal deaths from pregnancy until 6 weeks’ postpartum(PP) and, after 6 weeks PP in relation to the alive women living without HIV and women living with HIV

Of the 18 deaths recorded, three [3] occurred within pregnancy through to delivery up to 42 days PP, whilst fifteen [15] (including one case of road traffic accident) occurred post six weeks after delivery until the 5-year follow up time point. Within the WLWoH, there was only one fatality, a case of bladder cancer death recorded well beyond six weeks post-delivery resulting in a mortality frequency of 1/598: 0.17% (0.00-0.93) in this subgroup, Fig. 2.

### Comparing the deceased and the living in the UZBCS

**Fig. 3:**
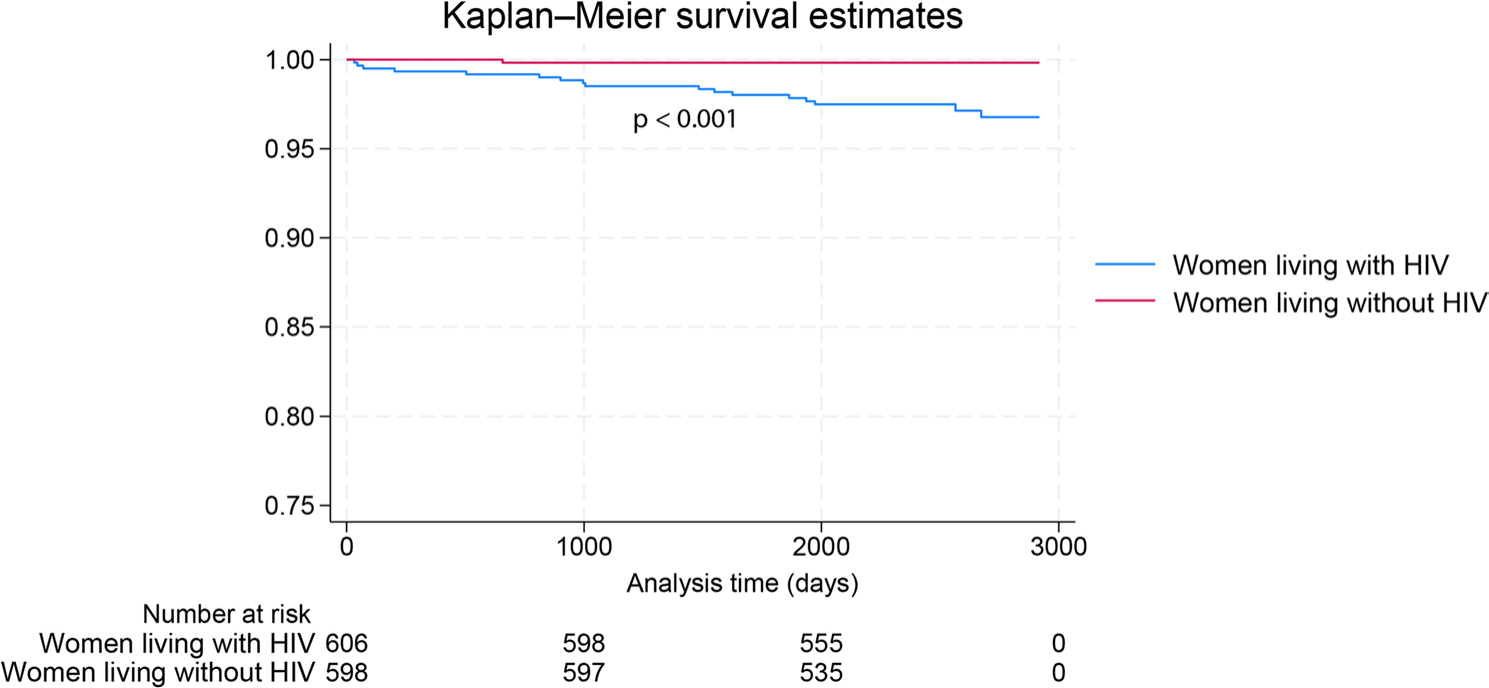
Kaplan-Meier survival estimates of women living without HIV and women living with HIV (15–49 years old). The Log rank test of equality of survival function, Chi = 13.9, *p* < 0.001

A univariate analysis, comparing the 18 deceased mothers against all 1190 alive women, no differences were observed as shown in Table 2. An HIV positive sero-status was the only significant factor associated with maternal death. Thus, WLWH were about 17 times more likely to die compared to their WLWoH peers OR (CI) 17.2 (2.3–123), *p* = 0.006, Table 2, and the Kaplan Meier survival estimates are shown in Fig. 3.

**Table 2:** Antenatal factors at enrolment associated with maternal mortality in the cohort participants (*N* = 1208), comparing the deceased (*N* = 18) against those alive (*N* = 1190)

Variable	Total *N* = 1208	Mother Deceased *N* = 18	Mother Alive *N* = 1190	Odds Ratio (95%CI)	*p*-Value
Maternal demographic characteristics					
Age in years	28.0 (24.0–33.0)	25.5 (22.0–33.0)	28.0 (24.0–33.0)	0.97 (0.90–1.05)	0.509
Education level					
Primary/None	62 (5.1)	1 (5.6)	61 (5.1)		
At least Sec	1146 (94.9)	17 (94.4)	1129 (94.9)	1.09 (0.14–8.32)	0.935
Marital status					
Married	1151 (95.3)	17 (94.4)	1134 (95.3)		0.866
Not-married	57 (4.7)	1 (5.6%)	56 (4.7)	1.19 (0.07–5.96)	
Employment status					
Unemployed	880 (72.9)	12 (66.7)	868 (72.9)		
Employed	328 (27.1)	6 (33.3)	332 (27.1)	0.74 (0.28–1.99)	0.554
Main religion					
Apostolic*	295 (26.1)	0 (0.0)	295 (26.5)		
Non-apostolic	835 (73.9)	18 (100.0)	817 (73.5)	Incalculable	0.061
Socio-economic and life style characteristics					
Money spend on food	60.00 (40.00-100.00)	70.00 (50.00-100.00)	60.00 (40.00-100.00)	1.00 (0.99–1.01)	0.839
Monthly family income	225.00 (141.50–339.00)	252.00 (150.00-345.50)	225.00 (141.00 −338.00)	0.9997 (0.9975–1.0020)	0.822
Current alcohol use					
Yes					
No	120 (9.9)	1 (5.6)	119 (10)	0.53 (0.03–2.61)	1.000
	1088 (90.1)	17 (94.4)	1071 (90)		
Smoking status					
Yes					1.000
No	3 (0.2)	0	3 (0.3)	Incalculable	
	1205 (97.8)	18 (100)	1187 (99.7)		
Obstetrics and health care related factors					
Parity	1.0 (1.0–2.0)	1.0 (0.0–2.0)	1.0 (1.0–2.0)	0.90 (0.60–1.35)	0.624
Gravida	3.0 (2.0–4.0)	2.5 (1.0–4.0)	3.0 (2.0–4.0)	0.87 (0.60–1.27)	0.475
Gestational age at enrolment	33.0 (29.9–36.1)	32.7 (30.0-33.3)	33.0 (29.9–36.1)	0.95 (0.86–1.05)	0.291
Spouse/intimate partner factors					
Spouse age difference	5.0 (3.0–8.0)	5.0 (3.0–7.0)	5.0 (3.0–9.0)	1.00 (0.90–1.10)	0.924
Partner tested for HIV					
No	243 (21.9)	5 (31.3)	238 (21.7)		
Yes	868 (78.1)	11 (68.7)	857 (78.3)	1.64 (0.56–4.76)	0.365
Spouse HIV status					
Positive	298 (34.5)	6 (54.5)	292 (34.2)		
Negative	567 (65.5)	5 (45.5)	562 (65.8)	2.31 (0.70–7.63)	0.170
Infections/double/triple coinfections					
Maternal HIV status					
Positive	608 (50.3)	17 (94.4)	591 (49.7)		
Negative	600 (49.7)	1 (5.6)	599 (50.3)	17.23 (2.29-129.88)	**0.006**
History tuberculosis infection					
Yes	58 (4.8)	2 (11.1)	56 (4.7)	2.53 (0.57–11.28)	0.223
No	1150 (95.2)	16 (88.9)	1134 (95.3)		
CMV IgM antibodies					
Present	85 (7.1)	2 (11.1)	83 (7.0)	1.66 (0.37–7.32)	0.507
Absent	1115 (92.9)	16 (88.9)	1099 (93.0)		
Syphilis antibodies					
Present	56 (4.6)	1 (5.6)	55 (4.6)		
Absent	1152 (95.4)	17 (94.4)	1135 (95.4)	1.21 (0.16–9.29)	0.852
Non-communicable conditions					
Anaemia					
Present	130 (10.8)	2 (11.1)	128 (10.8)		
Absent	1078 (89.2)	16 (88.9)	1062 (89.2)	1.04 (0.24–4.56)	0.962
Pedal oedema					
Present	111 (9.3)	2 (11.1)	109 (9.3)	1.22 (0.19–4.38)	0.680
Absent	1084 (90.7)	16 (88.9)	1068 (90.7)		
	(missing = 13)		(missing = 13)		
Blood pressure					
Hypotensive (< 90/<60)					
Normotensive					
(< 140&≥90/<90&≥60)	128 (10.6)	3 (16.7)	125 (10.5)	Incalculable	0.620
Hypertensive				Incalculable	
(≥ 140/≥90)	1048 (87.2)	15 (83.3)	1033 (87.2)		
	26 (2.2)	0	26 (2.2)		
	(missing = 6)		(missing = 6)		
Body mass index					
≤ 18.5	8 (0.7)	0	8 (0.7)	Incalculable	
> 18.5	1196 (99.3)	18 (100)	1178 (99.3)		1.000
	(missing = 4)		(missing = 4)		
Mid upper arm circumference (cm)					
≤ 23	88 (7.3)	1 (5.6)	87 (7.3)	1.34 (0.27–24.37)	1.000
> 23	1117 (92.7)	17 (94.4)	1100 (92.6)		
	(missing = 3)		(missing = 3)		

Data are expressed as n (%) or median (IQR); min-max unless stated otherwise

Statistical analysis: comparison of the 2 groups using the Kruskal Wallis test, Mann-Whitney U test or Fisher’s exact test where appropriate. *P*-values in bold font are statistically significant

^*^Health seeking behaviours differ by religion with the Apostolic sect shunning contemporary health care services, and believe in supernatural healing powers through faith

Up until 2-years after birth, the proportion of WLWH dying in our study was 0.50%.

### Cohort overall MMR

One thousand two hundred and two (1202) live births were recorded, 602 (including 9 set of twins) and 600 (4 including sets of twins) in the WLWH and WLWoH, respectively. Three [3] women died between pregnancy through up to 42 days PP. Thus, 3 direct maternal deaths were observed, one each in pregnancy, during childbirth and by 6 weeks PP. Consequently, the overall cohort MMR was at 3/1202; (CI) 249.6 (51.5-727.6)/100,000 live births. Seven [7] women presented with repeated pregnancies and their sociodemographic data were updated accordingly as second enrolment within the same cohort. The follow up rate at delivery was at 98.5%. Five more mothers were lost to follow up by 6 weeks PP.

### MMR by HIV status and characterising direct/pregnancy-related mortality

Within the WLWH sub-group, 591 (97.2%) were alive, with 3 (0.3%) direct maternal deaths recorded from pregnancy through to childbirth until 6 weeks PP, Fig. 2. By HIV status, among the WLWoH, MMR was incalculable as no death was observed versus three deaths recorded in WLWH; 3/602; 498.3 (102.9-1449.4)/100,000 live births. Thus, MMR in WLWH was almost twice as high compared to that of the overall cohort.

Of the 3 direct maternal deaths all occurring within the WLWH subgroup, the case of death in pregnancy presented with fever and painful lower limbs at the time of demise. Without an autopsy report for accrual cause of death it was not possible to ascertain whether the cause of death was sepsis related or was due to a deep vein thrombosis with pulmonary embolism. The cause of death for the second case was obstetric haemorrhage during childbirth following 5 cardiac arrests before hysterectomy for a retained placenta. The third fatality occurred within 42 days PP, a case of puerperal sepsis following a caesarean section. All these 3 preventable or treatable direct maternal deaths occurred at hospital facilities, and these constituted 17.6% of all the deaths recorded in the UZBCS.

### Comparing pregnancy factors of the deceased WLWH versus their randomly selected alive counterparts

In a univariate analysis comparing antenatal factors of each deceased mother to 4 randomly selected living counterparts within the WLWH subgroup, no differences were observed, Table 3. Again, there were no differences in the reported ART stock availabilities at the health centres, frequencies of ART-related side effects experienced nor whether ART doses were ever missed, Table 3. However, the deceased WLWH tended to be younger, median age (IQR) 25 (21.8–33.8**)** versus 31 (27-34.3) years in their alive counterparts, but borderline significance, *p* = 0.05. No differences were observed with respect to the relatively less sensitive WHO clinical staging between the two groups. Interestingly, 12.5% of the deceased mothers reported the presence of past TB infection and treatment, including the presence of a household member recently diagnosed with TB during the current pregnancy, Table 3. The deceased WLWH were not as immune competent; median CD4 + T-lymphocytes counts (IQR) 174 (101–286) cells/µL compared to 379 (264–494) cells/µL observed in their alive peers, *p* = 0.0009, Table 3. Up to 31.2% of the WLWH who later on died after delivery had not disclosed their HIV-status in pregnancy, compared to 11.2% in their alive peers, *p* = 0.014. Compared with their alive peers, dying in WLWH was associated with not being on ART, *p* = 0.038, Table 3. As expected, not being on ART was associated with higher HIV-RNA load; 75% of the deceased WLWH presented with unsuppressed HIV-RNA load (> 1000 copies per mL) compared to 18.8% in their alive peers, *p* = 0.0001, Table 3. Up to 40% the deceased were not aware of their spouses/intimate partners’ HIV status, Table 3.

**Table 3:** Baseline/pregnancy ≥ 20 weeks’ gestational age socio-demographic and life style characteristics, (co) infections, clinical data, spouse/intimate partner factors including HIV and antiretroviral therapy related factors in deceased (natural deaths) women living with HIV (WLWH) (*n* = 16) compared to their randomly selected alive peers (*n* = 64)

	Antenatal factors and risk of dying in WLWH			
	Overall (*N* = 80)	Deceased (*n* = 16)	Alive (*n* = 64)	*p*-value
Sociodemographic data				
Age in years				
Median (IQR)	30 (26-34.3)	25 (21.8–33.8)	31 (27-34.3)	0.052
Education level				
Primary/None	26 (32.5%)	5 (31.3%)	21 (32.8%)	1.000
At least Secondary level	54 (67.5%)	11 (68.7%)	43 (67.2%)	
Marital status				
Married	75 (93.8%)	15 (93.8%)	60 (93.8%)	1.000
Not married	5 (6.2%)	1 (6.2%)	4 (6.2%)	
Employment status				
Unemployed	62 (77.5%)	12 (75%)	50 (78.1%)	0.749
Employed	18 (22.5%)	4 (25%)	14 (21.9%)	
Current alcohol use				
Yes	11 (13.8%)	1 (6.2%)	10 (15.6%)	0.448
No	69 (86.2%)	15 (93.8%)	54 (84.4%)	
Spouse related factors				
Spouse age (years)				
Median (IQR)	37 (31 − 0)	33 (26-38.5)	37 (32–40)	0.067
Spouse occupation				
Employed	91 (94.7)	15 (100%)	56 (93.4%)	0.546
Unemployed	4 (5.3%)	0 (0%)	4 (6.6%)	
	(missing = 5)	(missing = 1)	(missing = 4)	
Spouse tested for HIV				
Don’t know	5 (6.3%)	1 (6.7%)	4 (6.3%)	0.166
No	14 (17.7%)	5 (33.3%)	9 (14%)	
Yes	60 (76%)	9 (60%)	51 (79.7%)	
	(missing = 1)	(missing = 1)		
Pregnancy related factors				
Gravida				
Median (IQR)	3 (2–4)	2 (1–3)	3(2–4)	0.068
Gestational age at first				
ANC booking (weeks)				
Median (IQR)	32.7 (28.7–35.3)	32.4 (29.6–33.1)	32.7 (28.7–36.2)	0.410
HIV related factors				
Disclosed HIV status?				
Yes	71 (88.8%)	11 (68.8%)	60 (93.8%)	**0.014**
No	9 (11.2%)	5 (31.2%)	4 (6.3%)	
Days since HIV				
diagnosis	409 (33.8–1726)	84.5 (0-2312.5)	514.5 (54.5–1623)	0.321
CD4 ^+^ T-lymphocyte count (cells/µL)				
Median (IQR)	344 (228–490)	174 (101–286)	379 (264–494)	**0.0009**
Viral load (copies/mL)				
≤50	40 (50%)	3 (18.8%)	37 (57.8%)	**0.0001**
51-1000	16 (20%)	1 (6.2%)	15 (23.4%)	
>1000	24 (30%)	12 (75%)	12 (18.8%)	
ART related factors				
Are you on ART?				
Yes	69 (86.3%)	11 (68.8%)	58 (90.6%)	**0.038**
No	11 (13.7%)	5 (31.2%)	6 (9.4%)	
Timing of ART initiation				
Post-conception	30 (37.5%)	4 (25%)	26 (40.6%)	0.094
Pre-conception	39 (48.7%)	7 (43.7%)	32 (50%)	
Naive	11 (13.8%)	5 (31.3%)	6 (9.4%)	
Ever missed ART dose?				
(*n* = 69 ART exposed only):				
Yes	7 (10.1%)	1 (9.1%)	6 (10.3%)	1.000
No	62 (89.9%)	10 (90.9%)	52 (89.7%)	
Days without ART				
following HIV diagnosis				
Median (IQR)	0 (0–30)	0 (0-173)	0 (0-186)	0.747
Duration of ART use				0.263
< 126 days	34 (42.5%)	9 (56.3%)	25 (39.1%)	
≥ 126 days	46 (57.5%)	7 (43.7%)	39 (60.9%)	
Duration of ART use				
≤ 365 days	46 (57.5%)	9 (56.3%)	37 (57.8%)	1.000
>365 days	34 (42.5%)	7 (43.7%)	27 (42.2%)	
ART side effects (*n* = 69):				
Present	19 (29.2%)	4 (36.4%)	15 (27.8%)	0.718
Absent	46 (70.8%)	7 (63.6%)	39 (72.2%)	
	(missing = 4)		(missing = 4)	
ART stock supply always available (*n* = 69):				
Yes	67 (97.1%)	10 (90.9%)	57 (98.3%)	0.295
No	2 (2.9%)	1 (9.1%)	1 (1.7%)	
(Co)Infections				
History of TB infection				
Yes	8 (10%)	2 (12.5%)	6 (9.4%)	0.657
No	72 (90%)	14 (87.5%)	58 (90.6%)	
TB treatment history				
Yes	8 (10%)	2 (12.5%)	6 (9.4%)	0.657
No	72 (90%)	14 (87.5%)	58 (90.6%)	
Was anyone diagnosed with TB in your household/living at your address during this pregnancy				
Yes	5 (6.3%)	2 (12.5%)	3 (4.7%)	0.260
No	75 (93.7%)	14 (87.5%)	61 (95.3%)	
Anti-CMV IgM				
Negative	75 (94.9%)	14 (87.5%)	61 (96.8%)	0.181
Positive	4 (5.1%)	2 (12.5%)	2 (3.2%)	
	(missing = 1)		(missing = 1)	
Syphilis antibodies				
Present	5 (6.3%)	1 (6.3%)	4 (9.4%)	1.000
Absent	75 (93.7%)	15 (93.7%)	60 (90.6%)	
Hepatitis b surface antigen				
Present	3 (3.8%)	2 (12.5%)	1 (1.6%)	0.100
Absent	77 (96.2%)	14 (87.5%)	63 (98.4%)	
Non-communicable conditions				
Anaemia, Hb ≤ 11 g/dL				
Present	40 (54.1%)	10 (66.7%)	30 (50.8%)	0.386
Absent	34 (45.9%) (missing = 6)	5 (33.3%)	29 (49.2%)	
		(missing = 1)	(missing = 5)	
Blood pressure				
Hypotensive (< 90/<60)	10 (12.7%)	2 (12.5%)	8 (12.7%)	1.000
Normotensive (< 140&≥90/<90&≥60)	69 (87.3%)	14 (87.5%)	55 (87.3%)	
Hypertensive (≥ 140/≥90)	0	0	0	
	(missing = 1)		(missing = 1)	
BMI				
≤18.5	1 (1.3%)	0	1 (1.6%)	1.000
>18.5	78 (98.7%)	16 (100%)	62 (98.4%)	
	(missing = 1)		(missing = 1)	
MUAC				
≤23 cm	7 (8.8%)	1 (6.3%)	6 (9.4%)	1.000
>23 cm	73 (91.2%)	15 (93.7%)	58 (90.6%)	
Perceived stress				
No	78 (97.5%)	16 (100%)	62 (96.9%)	1.000
Yes	2 (2.5%)	0	2 (3.1%)	
Perceived depression				
No	78 (98.7%)	16 (100%)	62 (98.4%)	1.000
Yes	1 (1.3%)	0	1 (1.6%)	
	(missing = 1)		(missing = 1)	

Data are expressed as n (%) or median (IQR); min-max unless stated otherwise

Statistical analysis: Comparisons between groups done using the Kruskal Wallis test, Mann-Whitney U test or Fisher’s exact test where appropriate. *P*-values in bold font are statistically significant

### Predictors of dying in WLHIV

In a regression model without variable elimination, a CD4 + T-lymphocytes counts of ≥ 350 cells/µL was protective, OR (95% CI): 6.3 (1.3–49.5), compared to WLWH with CD4 + T-lymphocytes counts of less than 350 cells/µL, *p* = 0.04. With the variable elimination model, the unsuppressed HIV-RNA load (> 1000 copies) increased the risk of dying, OR (95% CI): 5.2 (1.2–24.8), *p* = 0.03, compared to their alive peers who presented with suppressed HIV-RNA load of ≤ 1000 copies/mL, Table 4.

**Table 4:** Predictors of dying (natural deaths) or not dying in women living with HIV compared to their randomly selected alive peer regressions with and without variable elimination

	Predictors of maternal death in WLHIV					
	Without variable elimination			With variable elimination		
	OR	CI	*p*-value	OR	CI	*p*-value
Intercept	1.79	0.09–50.64	0.714	0.56	0.07–5.03	0.593
Disclosed your HIV status to anyone (yes)	0.13	0.01–1.56	0.121	0.29	0.04–1.80	0.188
Viral load (> 1000 copies/ml)	4.92	0.93–29.62	0.064	5.17	1.19–24.79	**0.031**
CD4^+^ T-lymphocyte count (≥ 350 cells/µl)	0.16	0.02–0.79	**0.040**	0.19	0.03–0.89	0.053
Gravida (per gravida)	0.63	0.31–1.15	0.149	-	-	-
Are you on ART (yes)	2.10	0.21–37.73	0.563	-	-	-
Days without ART after diagnosis (per day)	1.00	0.99–1.003	0.103	-	-	-

Statistical analysis: Logistic regression with and without variable elimination

## Discussion

### Summary of main findings

MMRs and the respective risk factors were reported in women of reproductive age in a resource limited setting where HIV burden is also high. The overall MMR in the UZBCS of pregnant women enrolled from 20 weeks’ gestational age was 249.6/100 000 live births. By HIV-status, MMR was 498.3/100 000 live births in WLWH, and these were 17 times more likely to die compared to WLWoH where no death cases were recorded by day 42 PP. WLWH with HIV-RNA load > 1000 copies/mL were 5 times more likely to die compared to their alive peers also living with HIV. Non-disclosure of HIV sero-status was associated with higher likelihoods of dying. The possible mechanism could have been poor adherence leading to suboptimal ART-exposure. In addition, a new HIV diagnosis, hence being ART-naïve was also a significant contributing factor.

There was a tendency of relatively younger WLWH dying, although this did not reach statistical significance, possibly because of the small numbers of deaths observed. In other studies, attaining higher educational qualification has been associated with older age at marriage and age at first pregnancy, leading to better economic opportunity, self-actualisation including empowerment, and consequently lower MMR [17]. However, in our study there was no evidence supporting this school of thought.

Up until 2 years after birth, the proportion of WLWH dying in our study was 0.50%, a figure much higher than the mortality ratio of 0.21% recorded in a related Harare Mabvuku Cohort of pregnant WLWH, also done during the same period, however, this study did not enrol controls of WLWoH [18]. Differences could be due follow up strategies and/or differences in the quality of health care services offered in these two communities with the later having better infrastructure, and probably offering better health care services.

The evidence of ART improving the quality of life for people living with HIV and prolonging their life expectancy is unquestionable, yet maternal HIV status still remains the most important determinant. In our study, WLWH were 17 more likely to die compared to WLWoH. Interestingly, this trend observed in the UZBCS mirrors that of the pre-ART era, the ZVITAMBO study that investigated the role of single dose vitamin A supplementation in the health of WLWH [19]. Furthermore, in the same study, similar to our findings, nearly all deaths were associated with TB coinfections.

In WLWH, HIV may increase mortality by accelerating HIV immunosuppression with attributable infections such as TB being the main cause of indirect deaths. Having HIV and worse still untreated or poorly controlled HIV increased the risk of women dying possibly due to both pregnant related ailments and opportunistic infections outside pregnancy. Our findings support the notion of other studies that lack of access to quality health care in SSA makes the indirect obstetric causes the main reason behind high deaths in women of reproductive age [20]. This observation warrants the need to integrate HIV services with other non-HIV health services such screening of key co-infections like TB testing and treatment from primary health care to district, provincial and national levels to reduce MMR. Infections would be reduced in pregnancy and after childbirth if early signs are detected and treated in a timely manner. Thus, these women in our study from relatively poorer high density areas may not have been receiving the healthcare they need, more so when up to 40% the deceased WLWH were not aware of their spouses/intimate partners’ HIV sero-status. In addition, up to 55.6% died at home. The fact that the majority of indirect cases occurred at home may point to the deteriorating economic climate affecting the health delivery system. In addition, one third of the deceased WLWH had not disclosed their sero-status, implying that stigma remains a challenge. Stigma surrounding HIV infection may affect the health seeking behaviours of WLWH as evidenced by the significant factors of not being on ART and non-disclosure of HIV status observed in our study. Non-disclosure of sero-status, hence not receiving adequate care and treatment to reduce the risk of women dying and/or vertically transmitting infections put the lives of the unborn children at risk.

As expected, suppressed HIV-RNA was protective. Determining markers of chronic immune activation would have shed a bit light on the 25% women with HIV-RNA ≤ 1000 copies per mL who nevertheless died. Markers of chronic immune activation are hallmarks of progressive HIV infections and may better predict disease outcome than plasma HIV RNA load.

In our study, all direct obstetric deaths occurred at central hospitals. In another pre-ART era study done at Harare Central Hospital, haemorrhage and puerperal sepsis were the main causes of direct maternal deaths, and this still remains true up to date [21]. This is not surprising since in Zimbabwe the top 3 causes of maternal mortality are obstetric haemorrhage, hypertensive disorders of pregnancy and sepsis [21]. A more recent central hospital based Harare study recorded a much higher case fatality ratio of 4.4% [22], compared to an overall mortality ratio of 1.4% observed in our study. As expected our mortality ratio was much lower. This difference could be because our study participants were at reduced risk by virtue of them being sampled from primary health centres.

The MMR in WLWH of 498/100,000 live births observed in the UZBCS, was much higher than the MMR of the recent national census of 2022 that was at 363/100,000 live births [9] and at 217/100,000 live birth in another study [7]. The figures are different probably due to differences in the methodologies and study populations included. Interestingly, the overall UZBCS MMR, combining both the WLWH and WLWoH was 249.6/100 000 live births, a figure falling within ranges of these two foregoing MMRs. In these previous studies, the HIV sero-status of the deceased women was not investigated or mentioned. Thus, inclusion of the maternal HIV status may be essential in future such studies.

The study period spanned the periods, before during and after the covid-19 pandemic making it unique. Interestingly, at least in the UZBCS the impact of the pandemic was minimal. Lockdowns and/or failures to access the usual health care at health institutions during the covid-19 era may have contributed to the much higher number of deaths that occurred at home. However, bigger and more robust studies are warranted to assess the impact of covid-19 on maternal mortality.

### The study strengths and limitations

Extensive comparative demographic and clinical characterisation of 608 WLWH and 600 WLWoH both sampled concurrently from the same community is the strength of our study. Thus, all research participants live in the same community with similar environmental exposures or conditions thus, an unusually homogenous study population. This enabled the comparison of the two groups alongside the traditional HIV-related factors of WLWH that died versus those of their alive counterparts. MMR was determined fairly accurate given the high follow up ratio by 6 weeks after delivery.

In our study, MMR in WLWoH was low, hence incalculable since no deaths were observed from pregnancy until 6 weeks PP. In addition, MMR was assessed only from at least 20 weeks’ gestational age. The covid-19 pandemic, and the challenging socio-economic environment left some research participants with no choice but to go back to the village, hence the mortality ratios observed after 6 weeks after delivery may be an under-estimate.

## Conclusion

MMR remains high in WLWH compared to WLWoH. WLWH were 17 times more likely to die compared to WLWoH. Non-disclosure of HIV sero-status was associated with the increased likelihoods of dying, probably due to poor adherence leading to suboptimal ART-exposure as depicted by unsuppressed HIV-RNA load. Encouraging sero-status disclosure by creating supportive environment for disclosure both at health facilities and at home remains essential. Thus, there is need to increase public awareness to reduce the stigma associated with HIV infection, in the process encouraging couple testing at the antenatal booking visits to reduce the proportion of non-disclosure as the nation works towards achieving the SDG target 3.1 by 2030.

A new HIV diagnosis, hence being ART-naïve was also a significant contributing factor. There is need to address barriers hindering HIV counselling and testing, treatment, adherence and continuation to suppress HIV-RNA load to reduce MMR in WLWH on lifelong ART.

## Supplementary Information

Supplementary Material 1.

## Data Availability

No datasets were generated or analysed during the current study.

## Abbreviations

ART: Antiretroviral therapy
BMI: Body mass index
CD4: Cluster of differentiation 4
CMV: Cytomegalovirus
Hb: Haemoglobin
HIV: Human Immunodeficiency virus
IgM: Immunoglobulin M
IQR: Interquartile Range
MUAC: Mid Upper Arm Circumference
TB: Tuberculosis
WHO: World Health Organisation
