# Supplementary material for: Maternal mortality ratio and deaths beyond 42 days up to 5-years post pregnancy in women living with HIV on life-long antiretroviral therapy in a resource limited setting
Source: BMC Pregnancy Childbirth. 2025 Nov 28;25:1274. doi: 10.1186/s12884-025-08151-5 (PMC12661766; doi:10.1186/s12884-025-08151-5)
Supplement: Supplementary file 1 — Supplementary Material 1. [file 12884_2025_8151_MOESM1_ESM.pdf]

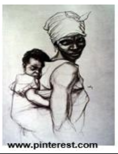

Participant ID ______

ENROLMENT MATERNAL DATA COLLECTION FORM

HIV EXPOSURE, DISEASE ACQUISITION AND PROGRESSION AMONG CHILDREN: ROLE OF MATERNAL IMMUNOGENETICS, VIRAL GENETIC DIVERSITY, HAART EXPOSURE, CO-MORBIDITIES AND PSYCHO-SOCIAL STATUS: THE UZ-CHS BIRTH COHORT

STUDY SITE ....

VISIT DATE (DD MM YY) ☐☐ ☐☐☐ ☐☐☐☐

START TIME (24hr notation) ☐☐:☐☐**Hrs**

Interviewer Initials: ________________________**Data Capturer 1 Initials:** __________

Data Capturer 2 Initials ____________________

---

1. **Date of Birth** *Makaberekwa riini?* ☐☐ ☐☐☐ ☐☐☐☐
2. **Para** ☐ **Gravida** ☐
3. **When was first date of your last menstrual period?** ☐☐ ☐☐☐ ☐☐☐☐
   Makapedzisira kugeza/kutevera riini (zuva rekutanga)?
4. **Gestational Age:** _________**weeks**
5. **EDD** ☐☐ ☐☐☐ ☐☐☐☐
6. **Documented Maternal HIV Status**
   ☐Negative
   ☐Positive
   If Positive, Specify **Date of Diagnosis** ☐☐ ☐☐☐ ☐☐☐☐
   Where____

A. SOCIO-DEMOGRAPHIC DATA Mibvunzo yakanangana nezvemagariro

1. **Tribe:** *Muri werudzi rupi?*

**Paternal** (*rwababa*)	**Maternal** (*rwaamai*)
0. ☐Manyika	☐Manyika
1. ☐Zezuru	☐Zezuru
2. ☐Karanga	☐Karanga
3. ☐Ndebele	☐Ndebele
4. ☐Non-Zimbabwean	☐Non-Zimbabwean
5. ☐Other (specify)	☐Other, (specify)

2. **Current Marital status:** *Pawanano makamira sei?*

0 ☐Single, never married/*Handina kumboroorwa*

1. ☐Married (Legal/Customary)/*Ndakaroorwa (kubvisirwa roora)*

2. ☐Married Polygamous

Participant ID ☐

3. ☐ Cohabiting/ *Kungogarisana (pasina kubvisirwa roora*)
4. ☐ Divorced/Separated/ *Ndakasiyana nemurume*
5. ☐ Widowed/ Ndakafirwa

If widowed (5.), cause of death:____
Kana vakashaya, vakashaya nei?

Date of Death *Vakashaya riini?* ☐☐ ☐☐☐ ☐☐☐

3. Spouse/Partner Age________Years
Murume wenyu/shamwari yepabonde ine makore mangani ekuberekwa?

4. Main Religion: *Muri vechitendero chipi?*

0. ☐ Apostolic/*Ndiri mupositori, wekwa*……………………………………………
1. ☐ Protestant/*Methodist/Anglican/Dutch Reformed*
2. ☐ Roman Catholic/*Ndiri MuRoma*
3. ☐ Pentecostal /*Ndiri muchechi yemweya*
4. ☐ African Traditionalist/*Ndinotenda muchivanhu chedu*
5. ☐ Islam/*Ndiri muChawa*
6. ☐ Other, **Specify**____

5. Educational Status: *Makadzidza kusvika gwaro ripi?*

0. ☐ None /*Handina kumbopinda chikoro*
1. ☐ Some Primary/*Handina kusvika giredi seveni*
2. ☐ Completed Primary/*Ndakapedza giredi seveni*
3. ☐ Some Secondary /*Ndakasvika kusekondari*
4. ☐ Completed secondary/*Ndakapedza sekondari*
5. ☐ Tertiary/*Ndakasvika kukoriji (state name)*___________________________

6. What is your current employment status? *Pari zvino muri kushanda here*?

1. ☐ Unemployed/*Handishandi*
2. ☐ Self-Employed/*Ndinozvishandira* ***Specify/Domayi zvamunoita***
____
3. ☐ Employed/*Ndinoshanda* **Specify**_______________________________________
4. ☐ Student/*Ndiri kudzidza* **Specify**_______________________________________

7. How much do you use on the following? (per month) *Pamwedzi munoshandisa marii pane zvinotevera?*

1. Rates and rentals? *Magetsi, mvura nerendi?* **US$**____________
2. Medical expenses? *Kurapwa nemishonga?* **US$**____________
3. Food? *Chikafu?* **US$**____________
4. School fees? *Mari yechikoro* **US$**____________

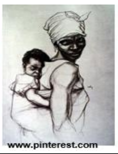

Participant ID [ ]

5. Other expenditure; *Zvimwewo zvingangodiwa*____________ **US$**_____________

8. What is your family <u>average</u> monthly income (combined)? US$_____________
Munowana marii pamwedzi wega wega semhuri? **(*should tally with amount stated in Question 7*)**

9. Did you or any household member <u>GO TO SLEEP AT NIGHT HUNGRY</u> because there was not enough food?
Imi kana mumwe wemumhuri pane here ***<u>akamborara</u>*** *asina kudya nekuti pakanga pasina chikafu chakakwana?*

0. ☐ No, Never
1. ☐ Rare (1-2 times/4 weeks)
2. ☐ Sometimes (3-10 times/ 4 weeks)
3. ☐ Often (>10 times in the past 4 weeks)

10. What is your <u>MAIN</u> source of energy for <u>cooking</u>?
Kana muchibika ***munowanzoshandisa*** *moto upi?*

1. ☐ Electricity/ *Magetsi*
2. ☐ Gas/ *Gasi*
3. ☐ Paraffin/ *Parafini*
4. ☐ Cooking Jelly/ *Jelli yekubikisa*
5. ☐ Coal/Charcoal/ *Marasha*
6. ☐ Wood/ *Huni*
7. ☐ Generator/ *Jenareta*
8. ☐ Others; *Zvimwewo* **Specify/** *Domai*____

11. Where do you cook from/place for cooking most of the time?
Munowanzobikira panzvimbo papi nguva zhinji?

1. ☐ Inside the house/ *Mumba*
2. ☐ In a separate building/structure/ *Muimwewo imba panze muchivanze chimwe chete*
3. ☐ Outdoors/ *Panze*
4. ☐ Other; *Pamwewo*; **Specify/** *Domai* ____

12. Do you own any of the following possessions/assets? *Mune zvinhu/midziyo inotevera? Tick appropriately*

Possessions/Assets	0-No	1-Yes	Is it Working/Intact?	
			0-No	1-Yes
**1.** Generator/*Jenareta*				
**2.** Solar Panel/*Sora panero*				
**3.** Radio/ *Wairesi*				
**4.** Television/TV/*Chivhitivhiti*				
**5.** Mobile phone/*Runhare-mbozha*				
**6.** Land line (phone)/*Foni yemumba*				

**7.** Refrigerator/*Firiji*				
**8.** Computer /*Kombiyuta*				
**9.** Bicycle/ *Bhasikoro/Bhizautare*				
**10.** Motorcycle/*Mudhudhudhu*				
**11.** Car/ *Motokari*				
**12.** Wheel borrow/ *Bhara*				
**13.** Piece of land (with deeds)/ *Munda*				
**14.** Farm animals/ *Zvipfuyo..................*				
**15.** Bank account/*Bhangi book/akaundi*				
**16.** Health insurance/Medical AID				

13. Ownership of residence in town

Muneimba here mudhorobha? 0. **No** ☐. 1. **Yes** ☐

If no go to Q15

14. If yes, how many rooms? *Kana munayo ine maruumu/mikamuri ma/mingani?*______________________*Go to Q16*

15. If No; *Kana musina imba*

1. ☐Renting *Yekurenda* ______ Rooms *Marumu*
2. ☐Inherited Family house/*Yenhaka* ______ Rooms *Marumu*
3. ☐Staying at a friend/relative's house for free ______________________Rooms/ *Marumu*
   Ndiri kugara pamba pehama/shamwari pachena/mahara

16. What's your household size (permanent members) **Total**___________

Muwanzogara muri vangani mumba menyu?

1. 0 - 5 years/*makore mashanu kana ari pasi* __________
2. 6 - 17 years/*makore matanhatu kusvika pamakore gumi nemanomwe* __________
3. 18+ years/*makore gumi nemasere zvichikwira* __________

17. How many rooms <u>do you use</u> in this house? __________________ **Rooms.**

Munoshandisa marumu mangani?

18. How many <u>families</u> live at your address? _______________ **Families.**

Imhuri ngani dzinogara pamba pamunogara?

19. Do you have a child with ill health/disabled? 0. **No**☐ 1. **Yes**☐

Mune mwana anorwara kana akaremera here?

If **Yes Specify** ____

20. Do you have an ID/Birth Certificate? 0. **No**☐ 1. **Yes**☐

Mune chitupa here/ kana tsamba yekuzvarwa kwenyu?

Alcohol, Smoking and Drugs Usage
Mibvunzo Yakanangana Nekunwa Kana Kuputa Zvinodhaka

21. Do you drink alcohol? 0. **No**☐ 1. **Yes**☐

Munonwa doro here? ***If No go to Q 24***

If <u>yes</u>, how often?

1. ☐Less than once/**Month**
2. ☐**Monthly**

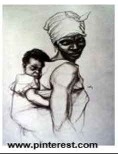

Participant ID ☐

3. ☐ 2-4 times/**Month**
4. ☐ 2-3 times/**Week**
5. ☐ 4 or more times/**Week**

22. Did you ever drink alcohol <u>during this pregnancy</u>? 0.**No** ☐ 1.**Yes** ☐

*Makambonwa zvinwiwa zvinodhaka here kubva **<u>zvamakabata pamuviri apa</u>**?*

If Yes; **Specify type alcoholic drink**____

Domai (ma)zita rechi(zvi)nwiwa

IF YES, **did you drink during pregnancy**: *Riinhi?*

First trimester:	0.**No** ☐	1.**Yes** ☐
Second trimester:	0.**No** ☐	1.**Yes** ☐
Third trimester:	0.**No** ☐	1.**Yes** ☐

23. How many <u>STANDARD</u> drinks containing alcohol do you have on a <u>typical day</u> drinking?

Munowanzonwa mabhodhoro /zvikari/ beer magi mangani pazuva?

		**<u>Daily</u>**	**<u>Weekly</u>**	**<u>Monthly</u>**
1.	☐ 1 or 2	☐	☐	☐
2.	☐ 3 or 4	☐	☐	☐
3.	☐ 5 or 6	☐	☐	☐
4.	☐ 7 or 9	☐	☐	☐
5.	☐ 10 or more	☐	☐	☐

24. Do you smoke cigarettes? **0. No** ☐ **1. Yes** ☐

Munoputa/bhema fodya here?

If yes, how many cigarettes you smoke during this pregnancy per day? / *Munoputa midzanga mingani pazuva kubva zvamakaita pamuviri apa?*________________________

25. Did you ever use the following drugs without being advised by a medical practitioner during this pregnancy?

Kubva zvamakaita pamuviri apa makambobvira mashandisa mishonga inotevera musina kurairidzwa naChiremba kana Mukoti?

1. Painkillers like **Stopayne**/**Pethidine**:	0.**No** ☐	1.**Yes** ☐
2. Cough syrups like **Broncleer**:	0.**No** ☐	1.**Yes** ☐
3. Sleeping tablets like Diazepam:	0.**No** ☐	1.**Yes** ☐

4. Others; **Specify:**____

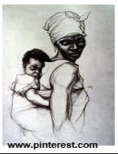

Participant ID ☐

B. WATER AND SANITATION
Mibvunzo inechekuita nemvura nekushandiswa kwezvimbuzi

1. **What is the type of toilet facility in your household?**
   Munoshandisa chimbuzi chakaita sei kumba kwenyu?.
   1. ☐ Inside flush to piped sewer system.
   2. ☐ Outside flush to piped sewer system
   3. ☐ Pit
   4. ☐ Blair latrine
   5. Others **specify**______

2. **How many households/<u>families</u> share this toilet facility?** ________________________
   <u>Imhuri</u> *ngani dzinoshandisa chimbuzi ichi?*

3. **What is your household <u>MAIN</u> water source for <u>drinking</u>?**
   Mvura ***<u>yekunwa</u>*** *yemhuri pamba yenyu munowanzoitora kupi?*
   1. ☐ Piped water into dwelling (**<u>Council</u>**)
   2. ☐ Public Borehole
   3. ☐ Protected dug well
   4. ☐ Bottled water (bought from shops)
   5. ☐ Others, **Specify** ____

4. **Do you treat your water before drinking?** 1. **Yes** ☐ 0.**No** ☐
   Mune zvamunoita kumvura musati mainwa here?
   If NO, go to question 5
   If yes, how? *Chii chamunoita kumvura isati yanwiwa?*
   1. ☐ Boiling
   2. ☐ Bleach/chlorine addition
   3. ☐ Solar disinfection
   4. ☐ Let it stand and settle
   5. ☐ Strain through a cloth
   6. ☐ Other **specify**____

5. **How many <u>days per week</u> do you have water running in your taps?** _______________
   Pasvondo munowana mvura mumatepi enyu mazuva mangani?

6. **How <u>often</u> does the sewer system around your home area overspill/bursts/blocks/clogs?**
   Kangani apo munomboona suweji yenyu yaka"bloka" ichirasira?
   - 0 ☐ Never/*Kana*
   - 1 ☐ Always/*Nguva dzose*
   - 2 ☐ At least once every month/ *Kangaita kamwe chete pamwedzi*
   - 3 ☐ At least once every year/ *Kangaita kamwe chete pagore*

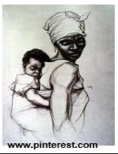

Participant ID ______

7. **Have you or other members of your household suffered from diarrheal diseases within the LAST MONTH?**
*Imi kana wemumhuri yenyu pane akamborwara nemanyoka here **mwedzi wapera**?*

0. No ☐ 1.**Yes** ☐

C. SPOUSE/PARTNER INVOLVEMENT
Mibvunzo ine chekuita nemurume wenyu /shamwari yepabonde

1. **Are you living together under the same roof** 0. **No** ☐ 1. **Yes** ☐
Imi nemurume wenyu /shamwari yepabonde munogara mese pamba pamwe chete here?
0 ☐ Always
1 ☐ Most of the time (3-5 days /week)
2 ☐ Sometimes (1-2 days per week)
3 ☐ Rarely (1-5 days per **month**)
4 ☐ Other, specify ____________________________

2. **Spouse's Occupation** *Murume wenyu /Shamwari yepabonde inoita basa rei?*
1. ☐ Unemployed
2. ☐ Self-Employed **Specify**________________**Industry**___________________
3. ☐ Employed **Specify**________________**Industry**___________________
4. ☐ Student **Specify**________________**Industry**___________________

3. **Has he been married before?** 0. **No** ☐ 1. **Yes** ☐
Murume wenyu/Shamwari yepabonde yakamboroora here musati maroorana?

4. **Since you got pregnant, have you experienced any marital/domestic violence?**
Kubva zvamakabata pamuviri makambosangana nedambudziko rekushungurudzwa here?
0. No ☐ *If no go to **Section D*** 1.**Yes** ☐

5. **If yes, Specify nature of violence**
*Kana makasangana naro tsanangudzai?*____

D. SEXUAL/REPRODUCTIVE HISTORY
MIBVUNZO INECHEKUITA NENYAYA DZEPABONDE?

1. **How old were you when you had your first sexual intercourse? _____YEARS**
Makange mavane makore mangani ekuberekwa pamakatanga nyaya dzepabonde?

2. **Do you have a casual sex partner(s)?** 0. **No** ☐ 1. **Yes** ☐
Mune imwe shamwari yepabonde asiri murume wenyu muridzi wepamuviri apa? If no go Q4

3. **Did you use condoms at your last sexual intercourse?** 0. **No** ☐ 1.**Yes** ☐
Makashandisa kondomu here pamakapedzisira bonde?

4. **What is the total number of life sexual partners to date?** __________
Kubva zvamakatanga bonde kusvikira pari nhasi; matamba bonde nevarume vangani?

5. **How many pregnancies have you had so far?** _______________
Pamuviri pechingani pamakatakura apa?

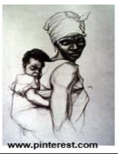

Participant ID ☐

6. **Have you ever used any vaginal drying/tightening/heating agent(s)?** *Munomboshandisa zvekupfeka pasikarudzi here muchigadzirira bonde?* **0. No** ☐ If No go to Q8 **1. Yes** ☐

7. **If yes, specify agent(s)/** *Domai zvamushandisa* ________________________________

   And <u>how often</u>? Kangani **1. <u>Daily</u>** ☐ **2. Often (<u>Weekly</u>)** ☐ **3. Rarely (<u>Monthly</u>)** ☐

8. **Have you ever used any method of family planning?**
   Makamboshandisa nzira dzekuronga mhuri here? **0. No** ☐ **1.Yes** ☐

9. **What method(s) of Family Planning were you using before this pregnancy?**
   Ndedzipi nzira dzokuronga mhuri dzamakamboshandisa musati mabata pamuviri apa?
   (Multiple answers possible)

   0. ☐ Natural
   1. ☐ Barrier (condom/diaphragm)
   2. ☐ Hormonal (pill/injection) **SPECIFY** ________________________________
   3. ☐ Implantable Devices
   4. ☐ Intra-uterine devices
   5. ☐ Emergency contraception
   6. ☐ Permanent methods

10. **Which method do you <u>intend</u> to use after delivery?** (*use same key as above)*___________
    Ndeipi nzira yamungada kuzoshandisa mushure mekunge masununguka?

11. **Why/<u>Main</u> Reason?** *<u>Sei muchifarira nzira iyi?</u>*
    ____

12. **How easy is it for you to get birth control methods?**
    Zviri nyore zvakadii kuwana nzira dzokuronga mhuri?

    1. ☐ Very Easy/ *Zviri nyore-nyore*
    2. ☐ Relatively easy/ *Zviri nyore*
    3. ☐ Sometimes difficulty/ *Dzimwe nguva zvakaoma*
    4. ☐ Very difficult/ *Zvakaoma chose*
    5. ☐ *Don't know/ Handizive*

13. **Has your spouse/current partner ever had /been:**
    Mumwe wenyu vakambo (ita):

1. A penile discharge? *Dhuropu?*	0. **No** ☐	1.**Yes** ☐	2. **Don't know** ☐
2. Genital warts? *Mhopo panhengo?*	0. **No** ☐	1.**Yes** ☐	2. **Don't know** ☐
3. Pain when passing out urine? *Kurwadziwa vachiita weti*	0. **No** ☐	1.**Yes** ☐	2. **Don't know** ☐
4. Circumcised? *Checheudzwa?*	0. **No** ☐	1.**Yes** ☐	2. **Don't know** ☐
5. Tested for HIV? *Ku"tetswa" HIV*	0. **No** ☐	1.**Yes** ☐	2. **Don't know** ☐

*If No or don't know to 5 go to **Section E***

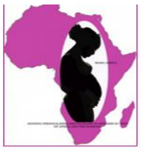

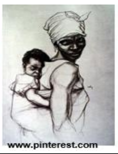

Participant ID [ ]

14. If yes, to HIV Testing, what was the test result?
Kana vakaongororwa/"kutetswa" HIV ma"rizautsi" akabuda sei?

0. Negative ☐ 1. **Positive** ☐ 2. **Don't know** ☐

*If negative go to **Section E***

15. Is he on ARV Treatment? 0. **No** ☐ 1. **Yes** ☐ 2. **Don't know** ☐
Vari pamushonga weHIV wemaARV here?

E. PLANNED FEEDING & NUTRITION
Mibvunzo inezvekuita nezvekudya

1. **How do you plan to feed the <u>baby</u> you are pregnant with <u>during the first 6 months of</u> birth?** *Hurongwa hwenyu hwakamira sei maringe nechikafu chamuchapa **<u>mwana</u>** wamakatakura kubva paanozvarwa kusvika ava nemwedzi mitanhatu?*

   1 ☐ Exclusive breast-feeding

   2 ☐ Mixed Feeding

   3 ☐ Formula milk/ alternative

   4 ☐ Other, specify ______________________________

2. **Why do you prefer this choice of feeding?** *Sei muchifarira urongwa ihwohwu?*

____

3. **If breastfeeding, how long do you plan to breastfeed?** ______________________**Months**
   Kana muchizoyamwisa, muri kutarisira kuyamwisa mwana uyu kusvikira akura kuita mwedzi mingani?

4. (*If respondent has/had other children; <u>Bvunza chete kana mai vakamboita mumwe mwana</u>.*)
   How long did you breastfeed your last child? _______________ **Months**
   Mwana wenyu mudikisa makamuyamwisa kusvikira ave nemwedzi mingani yekuberekwa?

5. **How many meals do <u>you</u> normally have per day as a <u>household</u>?** ____________________
   Munowanzodya kangani pazuva <u>semhuri</u>?

6. **How many meals do <u>you</u> normally have per day during this pregnancy?**_____________
   Makazvitakura kudai munowanzodya kangani pazuva? (PROBE MEAL)

7. **Did you eat anything today?** Pane chamadya here nhasi**? 0. No 1. Yes**
   If No, *Go to Question 9*

   If yes, what was the last time you ate something ? *(Time in Hours)*☐☐**:**☐☐**Hrs**
   Mapedzisira kudya nguvai nhasi ?

8. **What did you eat today <u>since you woke up, up to now?</u>**
   Kubva zvamamuka nhasi kusvikira parizvino madyei? Domai zvose zvamadya
   ____
   ____
   ____

9. **What did you eat in the last 24 hours, from the time you woke up yesterday to the time you went to bed?** *Kubva zvamakamuka nezuro muchiswera kusvikira pamakanorara makadyei? Domai chero chose chamakadya.*

   Breakfast ____

   In between ____

   Lunch ____

   In between ____

   Supper ____

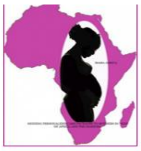

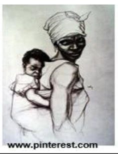

Participant ID |______|

10. IN GENERAL: how do you describe your appetite?
*(**Appetite**) Madiro (dyiro) enyu echikafu akamira sei?*

0. ☐ Good appetite, manages most of 3 meals/day
1. ☐ Poor appetite, manages more than half of meals provided)
2. ☐ Appetite null or virtually null or unable to eat (No food poor intake 1 meals/day)

11. How do you generally describe your appetite <u>during this pregnancy</u>?
*(**Appetite**) Mungati madyiro/madiro enyu echikafu akamira sei kamazvitakura kudai?*

1. ☐ Good appetite, manages most of 3 meals/day
2. ☐ Poor appetite, more than half of meals provided)
3. ☐ Appetite null or virtually null or unable to eat (No food poor intake 1 meals/day).

12. Did you or any other household member have to <u>EAT FEWER MEALS</u> in a day because there was <u>not enough food</u>?
Imi kana mumwe wemumhuri pane here akambodya kashoma (sekunge kamwe kana kaviri) pazuva nekuda kwekushayikwa kwechikafu?

0. ☐ No, Never
1. ☐ Rare (1-2 times/4 weeks)
2. ☐ Sometimes (3-10 times/ 4 weeks)
3. ☐ Often (>10 times in the past 4 weeks)

13. What are your current cravings in this pregnancy?
Munonyanya kuda/kufarira kudya chii nepamuviri penyu apa?
Specify/ *Domai*____

14. How often do you have the following foods/herbs/drinks during this pregnancy?
Munowanzodya rungani zvikafu/zvinwiwa zvinotevera? Tick appropriately

Food Type	Never	Daily	Weekly	Fortnightly	Monthly	Seasonal/ Rare
**Meats**<br>Beef/ *Nyama yemombe*						
Chicken/ *Nyama yehuku*						
Eggs/ *Mazai*						
Pork/ *Nyama yenguruve*						
Fish/ Hove)						
Kapenta/ Matemba						
Game/ *Nyama yemhuka dzesango*						
Madora/Macimbi						
Insects/ Traditional Ishwa, makurwe etc						
**Others (specify)**……………………………………………..						
**Grain/Cereals** Rapoko/ *Zviyo*						
Sorghum/ Mapfunde						
Millet/ *Mhunga*						
Sadza rechibage						
Rice/ *Mupunga*						
Whole grain cereals eg oats, bulgur						
**Others (specify)**…………………………………						
**Vegetables** (specify)<br>Beans						
Cow peas/ Nyemba						
Traditional vegetables – muboora, mutsine, munyevhe etc						

Participant ID ____________

Green leafy *covo, rape, spinachi, tsunga, etc*						
Cabbage						
Pumpkin/ *manhanga/mutikiti*						
Carrots						
Soya mince						
Mushroom/ *hova*						
Others (specify)………………………………..						
**Fruits** (specify type)<br>*Fill in in the appropriate column(s)*						
…………………………………………………						
………………………………………………….						
…………………………………………………						
………………………………………………….						
………………………………………………….						
**Fat/oils**<br>Nuts / *nzungu*						
Household butter/margarine use (kg)					……….kg	
Peanut butter / Dovi					………..ml	
Household refined cooking oil use					……..litres	
Other (specify)……………………						
**Fermented foods**<br>Yoghurt						
Cheese						
Mahewu						
Lacto						
Other (Specify)						
**Coffee**						
**Tea**						
**Herbs**<br>Turmeric						
Garlic						
Ginger						
Household salt use (grams)					……..grams	
Other spices specify)…………………………..						

15. How do you describe your ability to eat or/and retain food during this pregnancy?
Mungati munokwanisa kudya pasina kutambura kutsenga, kumedza kana kusvipurwa/kusvotwa muchizochaya? ***(Multiple responses possible)***

0. ☐ No difficulties in eating. No vomiting/diarrhoea
1. ☐ Vomiting/frequent regurgitation/mild diarrhoea
2. ☐ Difficulty swallowing
3. ☐ Problems with dentures or chewing affecting food intake
4. ☐ Severe vomiting and/or diarrhoea (>than 2 per day)

16. Have you been diagnosed with any digestive disease?
Makambobatwa here chero chirwere chemudumbu?

0. No ☐ **1. Yes** ☐ **If yes, specify** *Tick all that apply*

Condition	No	Yes	When diagnosed	Medication	Family History
1. Duodenal Ulcer?			☐☐ ☐☐☐ ☐☐☐☐		0.No ☐ 1.Yes ☐
2. Persistent epigastric pain/heartburn			☐☐ ☐☐☐ ☐☐☐☐		0.No ☐ 1.Yes ☐
3. Gastric Ulcer?			☐☐ ☐☐☐ ☐☐☐☐		0.No ☐ 1.Yes ☐
4. H. Pylori infection?			☐☐ ☐☐☐ ☐☐☐☐		0.No ☐ 1.Yes ☐
5. GI tract cancers? Specify…………………			☐☐ ☐☐☐ ☐☐☐☐		0.No ☐ 1.Yes ☐

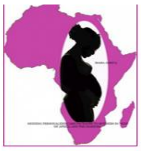

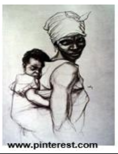

Participant ID ☐

6. **Others,** Specify…………………			☐☐ ☐☐☐ ☐☐☐☐		0.No ☐ 1.Yes ☐

17. What type of category of the <u>Bristol Stool Chart</u> would you allocate <u>your usual stool</u>? *Circle appropriately.*
Tsanangudza mamirire etsvina yamunowanzoita sekuti yakaomarara, yakaita manyoka?Tarisai mufanidzo munongedzere zvayakaita nguva zhinji (**Show respondent the actual chart**)

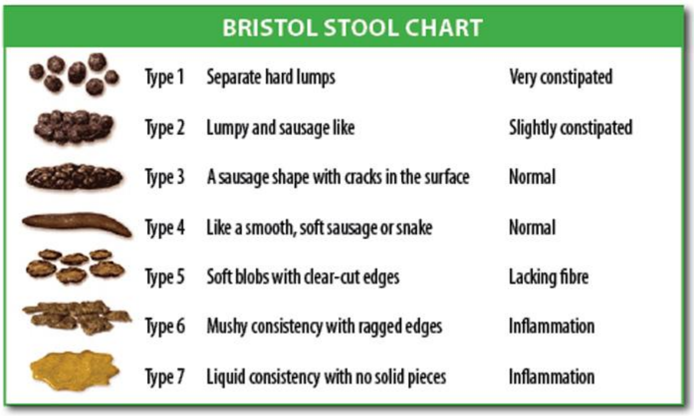

Cabot Health, Bristol Stool Chart http://www.cabothealth.com.

18. Indicate your current average daily stool frequency?
Munowanzoita tsvina kangani pazuva?

0. ☐ >/= Twice daily
1. ☐ Once daily
2. ☐ Once every 2 days
3. ☐ >Once every 2 days

F. GENERAL HEALTH AND MEDICAL HISTORY
Mibvunzo inezvekuita nezvehutano hwenyu

1. Assessment of Leisure Time Physical activity *Mabasa/mitambo inogwinyisa muviri*

Activity	Pre-pregnancy		First Trimester		Third Trimester	
	No ☐ Yes ☐	Hours/ Week	No ☐ Yes ☐	Hours/ Week	No ☐ Yes ☐	Hours/ Week
1. Recreational purposes Jog, walk	No ☐ Yes ☐		No ☐ Yes ☐		No ☐ Yes ☐	
2. Household/gardening	No ☐ Yes ☐		No ☐ Yes ☐		No ☐ Yes ☐	
3. Physical active transportation (walking)	No ☐ Yes ☐		No ☐ Yes ☐		No ☐ Yes ☐	
4. Physically active job	No ☐ Yes ☐		No ☐ Yes ☐		No ☐ Yes ☐	
5. Others	No ☐ Yes ☐		No ☐ Yes ☐		No ☐ Yes ☐	
6. Sleeping Patterns	☐ <6 hours<br>☐ 6-12 hours<br>☐ >12 hours		☐ <6 hours<br>☐ 6-12 hours<br>☐ >12 hours		☐ <6 hours<br>☐ 6-12 hours<br>☐ >12 hours	

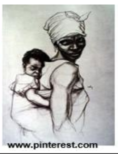

Participant ID ☐

2. What is your blood type?

Ropa renyu rinodaidzwa kuti irudzii? (I"taipi" ipi?)

1.	A+ ☐	A- ☐
2.	B+ ☐	B- ☐
3.	O+ ☐	O- ☐
4.	AB+ ☐	AB- ☐
5.	Don't know ☐	

3. Do you have any history of the following procedures/problems?

**History of the procedure/problem**	**Ever**	**Most Recent When**
Donate blood/ Kupa ropa	No ☐ Yes ☐	☐☐ ☐☐☐ ☐☐☐☐
Blood transfusion/ Kupiwa/wedzerwa ropa here?	No ☐ Yes ☐	☐☐ ☐☐☐ ☐☐☐☐
Pap smear *Makamboongororwa here zvegomarara remuromo wechibereko*	No ☐ Yes ☐	☐☐ ☐☐☐ ☐☐☐☐
Fracture/ Kutyoka bhonzo	No ☐ Yes ☐	☐☐ ☐☐☐ ☐☐☐☐
Anemia/ Kushaya ropa rakakwana	No ☐ Yes ☐	☐☐ ☐☐☐ ☐☐☐☐
Tattoo/ Nyora	No ☐ Yes ☐	☐☐ ☐☐☐ ☐☐☐☐

4. Complete the following TB symptoms checklist. Mark 'yes' if <u>you</u> have experienced any of listed symptoms.

Taurai kuti hongu kana kuti kwete pane mibvunzo inotevera ine chekuita nezvechirwere chorurindi (TB)

New, **productive cough** for more than 2 weeks? *Makambokosora muchibuditsa gararwa here kwenguva inodarika masvondo maviri?*	No ☐	Yes ☐
Hoarseness lasting more than 3 weeks? *Makamboshoshoma izwi here kwenguva inodarika masvondo matatu?*	No ☐	Yes ☐
Hemoptysis, Coughing up blood? *Makambokosora ropa here?*	No ☐	Yes ☐
<u>Night sweats</u> lasting more than 1 week? *Makambodikitira makarara here kwesvondo rose kana kupfuura?*	No ☐	Yes ☐
Unintentional <u>weight loss</u> (≥10%) over the past 2 months? *Makambopera muviri zvamusinganzwisisi mumwedzi miviri yadarika?*	No ☐	Yes ☐
Unusually tired over the past 3 weeks? *Makambonzwa kuneta zvakakanyanya mumasvondo matatu apfuura?*	No ☐	Yes ☐
Fever lasting more than 1 week? *Makambonzwa muviri kupisa kwesvondo rose kana kupfuura?*	No ☐	Yes ☐
Chills lasting more than 1 week? *Makambonzwa kupindwa nechando mumuviri kwesvondo rose kana kupfuura?*	No ☐	Yes ☐
Enlarged cervical lymph nodes. *Makamboita mambabvu epahuro here?*	No ☐	Yes ☐
<u>Is anyone</u> having severe / prolonged cough in the household/living at your address during this pregnancy? *Pane wemumhuri kana vamuri kugara navo pamba pamunogara vari kukosora zvakanyanya kukwenguva yakareba here pari zvino?*	No ☐	Yes ☐
Was anyone diagnosed TB in household/ living at your address during the <u>past 12 months</u>? *Pane wemumhuri kana vamuri kugara navo pamba pamunogara vakabatwa chirwere cheTB here <u>mugore rapfuura</u>?*	No ☐	Yes ☐

Participant ID ☐

5. **Previous TB Testing 0. No** ☐ **1. Yes** ☐ **if yes,** Date and results of previous Tb test:
Kana makambobatwa chirwere cherurindi, itetsi ipi yamakaitwa, rinhi?

**TB Test**	**Done**	**Result (P/N)**	**Date**
Sputum	No☐ Yes☐	____________	☐☐ ☐☐☐ ☐☐☐☐
Tuberculin skin test (TST)	No☐ Yes☐	____________	☐☐ ☐☐☐ ☐☐☐☐
Chest x-ray	No☐ Yes☐	____________	☐☐ ☐☐☐ ☐☐☐☐
IGRA	No☐ Yes☐	____________	☐☐ ☐☐☐ ☐☐☐☐

6. **Any history of treatment of TB infection or disease**:
Makamborapwa cherwere cherurindi? 0.No ☐ 1.Yes ☐
If No, go to question 14

7. **IF Yes, state when:** *Kana makamborapwa chirwere cheTB huye riini?*

8. **Date of drug start** ☐☐ ☐☐☐ ☐☐☐☐
Makatanga riini kunwa mushonga?

9. **Date completed** ☐☐ ☐☐☐ ☐☐☐☐
Makapedza kunwa mushonga riini?

10. **Medication received**: ____
Mungaziva kuti mainwa/kubayiwa mushonga unonzi chii?

11. **Completed Prescribed Course:** 0.No ☐ 1.Yes ☐
Makapedza course yemushonga yacho yose here?

12. **History of prior exposure to someone with TB disease:** 0.No ☐ 1.Yes ☐
Makambogara/kushanda nemunhu airwara neTB here?

13. **Where** *Kupi?* ____________________
Date *Riini?* ☐☐ ☐☐☐ ☐☐☐☐

14. **Have you ever been told by a doctor or health professional that you had any of the following infections? Check source document**
Makambobatwa zvirwere zvinotevera here kuchipatara kana kukiriniki?

**Illness**	**No**	**Yes**	**When diagnosed**	**Medication**	**Any household member with similar infection**
1) TB/ *Rurindi*			☐☐ ☐☐☐ ☐☐☐☐		No☐ Yes☐
2) Pneumonia *Mabayo*			☐☐ ☐☐☐ ☐☐☐☐		No☐ Yes☐
3) Candida/trash			☐☐ ☐☐☐ ☐☐☐☐		No☐ Yes☐
4) Cryptococcal			☐☐ ☐☐☐ ☐☐☐☐		No☐ Yes☐
5) Malaria *marariya*			☐☐ ☐☐☐ ☐☐☐☐		No☐ Yes☐
6) Schistosoma *Biriharazia*			☐☐ ☐☐☐ ☐☐☐☐		No☐ Yes☐

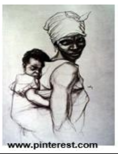

Participant ID ☐

7) Tinea infections *Zvisasa*			☐☐ ☐☐☐ ☐☐☐☐		No ☐ Yes ☐
8) Intenstinal worms Makonye mudumbu			☐☐ ☐☐☐ ☐☐☐☐		No ☐ Yes ☐
9) Syphillis *Njovhera*			☐☐ ☐☐☐ ☐☐☐☐		No ☐ Yes ☐
10) Eye infections			☐☐ ☐☐☐ ☐☐☐☐		No ☐ Yes ☐
Other, **Infections** Specify……………….			☐☐ ☐☐☐ ☐☐☐☐		No ☐ Yes ☐

15. Any <u>HISTORY/CURRENT</u> infections of the following <u>during this pregnancy</u>?
(*Check source document,* *Confirm from the ANC card***)**

**Infection**	**No/Yes**	**Treated**	**Partner treated**	**When**
Pelvic inflammatory diseases	No ☐ Yes ☐	No ☐ Yes ☐	No ☐ Yes ☐	☐☐ ☐☐☐ ☐☐☐☐
Pelvic abscess	No ☐ Yes ☐	No ☐ Yes ☐	No ☐ Yes ☐	☐☐ ☐☐☐ ☐☐☐☐
Abnormal vaginal discharges	No ☐ Yes ☐	No ☐ Yes ☐	No ☐ Yes ☐	☐☐ ☐☐☐ ☐☐☐☐
Pain/ burning sensation on urination	No ☐ Yes ☐	No ☐ Yes ☐	No ☐ Yes ☐	☐☐ ☐☐☐ ☐☐☐☐
Genital warts	No ☐ Yes ☐	No ☐ Yes ☐	No ☐ Yes ☐	☐☐ ☐☐☐ ☐☐☐☐
Vulva itchiness	No ☐ Yes ☐	No ☐ Yes ☐	No ☐ Yes ☐	☐☐ ☐☐☐ ☐☐☐☐
Genital sores	No ☐ Yes ☐	No ☐ Yes ☐	No ☐ Yes ☐	☐☐ ☐☐☐ ☐☐☐☐

16. Have you ever been told by a doctor or health professional that you had any of the following <u>conditions?</u> <u>*Makambobatwa here zvirwere zvinotevera kuchipatara?*</u>

**Condition**	**Yes**	**No**	**When diagnosed**	**Medication**	**Family History**
Hypertension (*BP)*			☐☐ ☐☐☐ ☐☐☐☐		No ☐ Yes ☐
Stress, *Kufungisisa zvakanyanya*			☐☐ ☐☐☐ ☐☐☐☐		No ☐ Yes ☐
Depression *kushushikana pfungwa*			☐☐ ☐☐☐ ☐☐☐☐		No ☐ Yes ☐
Persistent headache *Musoro unogarotema*			☐☐ ☐☐☐ ☐☐☐☐		No ☐ Yes ☐
Persistent nose bleeding Kubuda mututu			☐☐ ☐☐☐ ☐☐☐☐		No ☐ Yes ☐
Persistent vaginal bleeding Kuramba uchibuda ropa kuzasi			☐☐ ☐☐☐ ☐☐☐☐		No ☐ Yes ☐
Back pain/Musana			☐☐ ☐☐☐ ☐☐☐☐		No ☐ Yes ☐
Visual disturbances *Kusaona zvakanaka*			☐☐ ☐☐☐ ☐☐☐☐		No ☐ Yes ☐
Diabetes / *Shuga*			☐☐ ☐☐☐ ☐☐☐☐		No ☐ Yes ☐
Heart disease *Chirwere chemwoyo*			☐☐ ☐☐☐ ☐☐☐☐		No ☐ Yes ☐
Stroke "Sturoko"/ *Kuoma mutezo*			☐☐ ☐☐☐ ☐☐☐☐		No ☐ Yes ☐
Gout			☐☐ ☐☐☐ ☐☐☐☐		No ☐ Yes ☐

Participant ID

Renal failure, *chirwere cheitsvo*			☐☐ ☐☐☐ ☐☐☐☐		No☐Yes☐
Hay-fever			☐☐ ☐☐☐ ☐☐☐☐		No☐Yes☐
Asthma /*asima*			☐☐ ☐☐☐ ☐☐☐☐		No☐Yes☐
Eczema			☐☐ ☐☐☐ ☐☐☐☐		No☐Yes☐
Food allergies *Chikafu chamusingawirirani Nacho/kukurwarisai Specify……………………………*			☐☐ ☐☐☐ ☐☐☐☐		No☐Yes☐
Joint pains/arthritis *Chirwere chemabonzo*			☐☐ ☐☐☐ ☐☐☐☐		No☐Yes☐
Cancer/ *Gomarara*			☐☐ ☐☐☐ ☐☐☐☐		No☐Yes☐
Epilepsy/ *Pfari/kugwina*			☐☐ ☐☐☐ ☐☐☐☐		No☐Yes☐
Underweight/ *Kuondoroka*			☐☐ ☐☐☐ ☐☐☐☐		No☐Yes☐
Overweight/ *Kufutisa*			☐☐ ☐☐☐ ☐☐☐☐		No☐Yes☐
Toothache/ *Zino*			☐☐ ☐☐☐ ☐☐☐☐		No☐Yes☐
Tooth extraction/ *Kudzurwa zino*			☐☐ ☐☐☐ ☐☐☐☐		No☐Yes☐
Gum problems/ *Dambudziko nematadza*			☐☐ ☐☐☐ ☐☐☐☐		No☐Yes☐
Mental health problem/ *Chirwere chepfungwa*			☐☐ ☐☐☐ ☐☐☐☐		No☐Yes☐
Any form of disability/ *Kuremara*			☐☐ ☐☐☐ ☐☐☐☐		No☐Yes☐
Other……………………			☐☐ ☐☐☐ ☐☐☐☐		No☐Yes☐

G. OBSTETRIC HISTORY & CURRENT PREGNANCY
Mibvunzo yenhoroondo dzepamuviri

1. **What was your age at first pregnancy? ______________years**
   Makange muine makore mangani pamakabata pamuviri pekutanga?

2. **When did you know that you were pregnant with this pregnancy? ___________months**
   *Makazoziva kuti makazvitakura pamuviri apa pave ne**mwedzi** mingani?*

3. **Was this pregnancy planned? (***If unplanned***, PROBE for reason)**
   Makanga makaronga here pamuviri apa?
   1. ☐Planned
   2. ☐Unplanned, it just happened/a mistake
   3. ☐Forced looking for a boy/girl child
   4. ☐Strengthen marriage/relationship
   5. ☐**Other specify……………………………….**

4. **Do you intend to get pregnant in future?** 0. **No**☐ 1.**Yes** ☐
   Mune tarisiro yekuita pamuviri zvakare here mune ramangwana renyu?
   If yes, when?______________________year(s)
   Kana tarisiro iripo, riinhi, tingati mumakore mangani kubva nhasi?

   If yes, how many children do you plan/intend to have? __________________
   Muri kutarisira kubereka/kuzvara vana vangani?

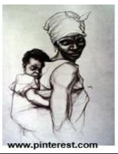

Participant ID ☐

5. Who makes the final decision on the number of children you intend to have?
Ndiyani anoronga nekukuudzayi kuti munofanira kuita vana vangani?

0. ☐ Self
1. ☐ Husband/Spouse/Partner
2. ☐ Both
3. ☐ Other (Specify) ______________________________

6. How many pregnancies have you ever had (regardless of its outcome)? __________
Makaita mimba ngani (chero mimba tisingaverengi kana makazobata mwana kana kuti kwete)?
Ask and tick appropriately

***Mode of Delivery codes** : **1.** NVD **2** C-Section, **3**-Breach Extraction **4** Vacuum Extraction **5.** Forceps*
***Pregnancy Outcome codes: 1.** Live Birth <u>Pre-term</u> **2.**Live Birth Full <u>Term</u> **3.** Still Birth **4.** Abortion/Miscarriage*
Sex codes: **Girl (G), Boy (B)**

Preg. No	Year	Pregnancy Outcome	Mode of Delivery	Sex	Health Centre / Place of delivery	Any Complications	Partner involvement In pregnancy	Child Breastfed	Child alive
1							☐No ☐Yes	☐No ☐Yes	☐No ☐Yes
2							☐No ☐Yes	☐No ☐Yes	☐No ☐Yes
3							☐No ☐Yes	☐No ☐Yes	☐No ☐Yes
4							☐No ☐Yes	☐No ☐Yes	☐No ☐Yes
5							☐No ☐Yes	☐No ☐Yes	☐No ☐Yes
6							☐No ☐Yes	☐No ☐Yes	☐No ☐Yes
7							☐No ☐Yes	☐No ☐Yes	☐No ☐Yes
8							☐No ☐Yes	☐No ☐Yes	☐No ☐Yes

7. Do <u>ALL</u> your children/pregnancies share the same father? 0. **No**☐ 1. **Yes**☐ 2. **N/A**☐
Vana venyu vose nepamuviri apa vana baba mumwe chete here? NOT APPLICABLE FOR FIRST PREGNANCY

8. At what stage in your pregnancy did you register your current pregnancy at the health centre? *Makanyoresa nhumbu yenyu yavane mwedzi mingani?*__________________

9. Have you missed any ANC of the visits? 0. **No**☐ 1. **Yes** ☐
Pane pamakambokundikana kuenda kunoonekwa here kusikero? If yes, why
____

10. What are the danger signs/complications of pregnancy that you must look out for and immediately visit a health facility?
Ndezvepi zvamungangoti makanzwa kana kuona zvingangokanganisa pamuviri zvinoita kuti mumhanye kuchipatara nekukurumidza?
List/Domai____
____

11. Have you ever had a scan done in this pregnancy? 0. **No**☐. 1. **Yes**☐
Makamboitwa scan here yepamuviri apa?

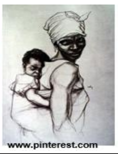

Participant ID ☐

12. If yes, When? *Kana makaitwa scan, yakaitwa rini* ☐☐ ☐☐☐ ☐☐☐☐

Why; *sei scan yakaitwa?*______________________

13. Have you received any vaccination during this pregnancy? 0.**No** ☐ 1. **Yes** ☐

Mune nhomba here yamakabayiwa makazvitakura kudai?

14. If yes, please give the name of the vaccine________________________________

Kana makabayiwa, domai zita renhomba

Date of last dose ☐☐ ☐☐☐ ☐☐☐☐ *Confirm from the ANC card*

Kana makabayiwa munga/yeuka/tondera kuti zvakaitika riini?

15. How has been your health since you got pregnant?

Hutano hwenyu hwakamira sei kubva zvamakaita pamuviri?

0. ☐ Very good, healthy, *Ndakasimba chaizvo*
1. ☐ Sick, but treated from home *Kungorwarawo zvekurapika kumba*
2. ☐ Just mood swing and morning sickness, nothing to worry about *Zvekungo"mudhura"/ku"bhoekana", nekungorutsa mangwanani chete*
3. ☐ Not well, been visiting the clinic now and again *Kusanzwa zvakanaka ndichingoenda-enda kukiriniki*

16. Have you ever been hospitalized during this pregnancy?

Makambopihwa mubhedha muchipatara here kubva zvamakabata pamuviri?

0. **No** ☐ *If no go Q18* 1. **Yes** ☐

17. If yes WHY? *Kana makapihwa mubhedha mairwara nei?*

**Illness Chirwere**	**Admission Where (health centre) *Kupi***	**How many times? *Kangani***	**How long? (days) *Mazuva mangani***

18. CURRENT MEDICATIONS

Mishonga yamuri kunwa pari zvino

**Medication (Fill in appropriately)**	**No**	**Yes**	**Start Date DD/MM/YY**	**Prescribed by?**
1. Folate supplementation				
2. Zinc supplementation				
3. Iron tablets				
4. Anti-acid				
5. Multivitamins				
6. Anti-biotic(s)*				
7. Antimalarial				
8. ARVs				
9. Cotrimoxazole				
10. **Others**............................				
11. Traditional /Chinese herbs				
12. /Specify name of herb?<br>Purpose?<br>How often you take it?<br>Any Side Effects?	______________<br>______________<br>______________<br>______________			

FINISH TIME: (24hr notation) ☐☐:☐☐**Hrs**

END OF QUESTIONNAIRE FOR THE <u>HIV NEGATIVES</u>

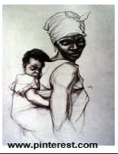

Participant ID ☐

HIV POSITIVE MOTHERS ONLY

1. **Have you disclosed your HIV status to anyone? 0. No** ☐ **1. Yes** ☐
If no, go to Q3.
Pane munhu wamakamboudza kuti mune hutachiwana hwe HIV here?
Kana vasina kuudza munhu endai paQ3

2. **If yes, who did you disclose your HIV status to (relationship to you)?** Go to Q4
Kana aripo vamakaudza, ndiyani wenyu?
1. ________________________ 2. ____________________ 3. __________________

3. **If No, do you intend to disclose your HIV status in the future?** 0. **No** ☐ 1. **Yes** ☐
Mune tarisiro yekubuda pachena kuti mune hutachiona weHIV mune remangwana?

4. **When did you/do you intend to disclose your HIV Status?**
Maka(mungango)buda pachena kwapera nguva yakadii mave kuziva mamiriro enyu?
☐ Immediately after testing positive (within 1 week)
☐ Within six months
☐ Within one year
☐ 1-2 years
☐ > 2 years

5. **Do you at any time feel stigmatized because of your HIV status?**
Pane pamunombonzwa sekunge muri kusemwa nekuda kwekuti muneHIV? If yes, probe more
0 ☐ Not at all, *Kana, hazvimboitika*
1 ☐ Rarely; *Nedzimwewo nguva dziri kure*
2 ☐ Quite often, *Zvinoitika kakawanda*
3 ☐ Always *Zvinoitikika nguva dzose*

6. **Are you on ART**? 0. No ☐ 1. Yes ☐
Muri kunwa mapiritsi anoderedza HIV mumuviri here, maARVs?
If NOT, Why? ____
Kana musinganwi, sei musirikunwa?
If yes, when did you start? Specify date ☐☐ ☐☐☐ ☐☐☐☐
Kana muchinwa, makatanga riini?

7. **Specify current drug regimen**:

		**Date you took your previous dose**	**Time you took your previous dose**
**i)**	☐ Tenolam -E	☐☐ ☐☐☐ ☐☐☐☐	__________
**ii)**	☐ Tenolam-N	☐☐ ☐☐☐ ☐☐☐☐	__________
**iii)**	☐ Other Specify ____________________		

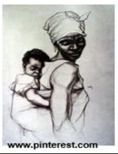

Participant ID ☐

8. Have you ever switched/changed your ARV regimens? 0. **No** ☐ 1. **Yes** ☐
Makambochinja here mapiritisi emaARV amurikunwa?

If yes specify

From (Drug):	TO (Drug):	Date
**1.**		☐☐ ☐☐☐ ☐☐☐☐
**2.**		☐☐ ☐☐☐ ☐☐☐☐
**3.**		☐☐ ☐☐☐ ☐☐☐☐

9. Any MAIN side effect experienced? ____________________________________
Pane zvamunonzwa here zvisinga kuitirei zvakanaka mushure mekunge manwa maARV?

10. Do you always have a constant supply of your ARV? 0. **No** ☐ 1. **Yes** ☐
Munowana maARVs aya nguva dzose?

If no, why ____
Kana musingamawani nguva dzose, sei achimboshayikwa?

11. In the past week, how many days have you missed taking all your doses?
Pasvondo rapfuura mazuva mangani amusina kunwa mapiritsi enyu sezvamunotarisirwa?

0. ☐ None
1. ☐ 1-2 days
2. ☐ 3-4 days
3. ☐ >5 days

12. If none to Q11, when was the last time you ever missed your ARV? ______________
Ndiriini pamusina kunwa mapiritsi enyu?

13. Why did you miss? *Sei musina kunwa* ________________________________

14. Confirm if the following tests were performed within the past year? *Ndokumbira kuziwa kuti makaitwa ongororo dzipi gore rapfura?* ***Confirm from any source documents***

Test	Yes	No	Date test done	Site/Institution	Test Result
HIV Test			☐☐ ☐☐☐ ☐☐☐☐		
CD4 Count			☐☐ ☐☐☐ ☐☐☐☐		
FBC			☐☐ ☐☐☐ ☐☐☐☐		
U&Es			☐☐ ☐☐☐ ☐☐☐☐		
LFTs			☐☐ ☐☐☐ ☐☐☐☐		
Syphilis			☐☐ ☐☐☐ ☐☐☐☐		
Viral load			☐☐ ☐☐☐ ☐☐☐☐		
Drug Resistance Test			☐☐ ☐☐☐ ☐☐☐☐		

FINISH TIME: (24hr notation) ☐☐:☐☐**Hrs**

END OF QUESTIONNAIRE
